# Immunoinformatics-based design of a multi-epitope mRNA vaccine candidate targeting the major human-infecting hepatitis E virus genotypes 1–4

**DOI:** 10.3389/fbinf.2026.1922523

**Published:** 2026-09-23

**Authors:** Maham Mehrangaze Khonza, Zain Saeed, Zohra Jabeen, Wafaa Arab, Sehrish Kakakhel, Maham Yamin, Mazen M. Ghaith, Aneela Javed, Ali Zohaib, Rwaa H. Abdulal

**Affiliations:** 1 Atta-Ur-Rahman School of Applied Biosciences, National University of Sciences and Technology, Islamabad, Pakistan; 2 Vaccines and Immunotherapy Unit, King Fahd Medical Research Center, King Abdulaziz University, Jeddah, Saudi Arabia; 3 Department of Biochemistry, Abdul Wali Khan University Mardan, Mardan, Pakistan; 4 Department of Microbiology and Immunology, Peter Doherty Institute for Infection and Immunity, The University of Melbourne, Melbourne, VIC, Australia; 5 Department of Clinical Laboratory Sciences, Faculty of Applied Medical Sciences, Umm Al‐Qura University, Makkah, Saudi Arabia; 6 Infectious Disease Research Department, King Abdullah International Medical Research Center, Jeddah, Saudi Arabia; 7 King Saud bin Abdulaziz University for Health Sciences, Jeddah, Saudi Arabia; 8 Ministry of the National Guard - Health Affairs, Jeddah, Saudi Arabia

**Keywords:** hepatitis E virus, immunoinformatics, mRNA vaccine, multi-epitope mRNA vaccine, ORF2, reverse vaccinology, vaccine design, viral genotypes

## Abstract

**Background:**

Hepatitis E virus (HEV) is one of the leading causes of acute and chronic hepatitis among humans across the globe and is linked to severe diseases in immunocompromised individuals and pregnant women. Despite its substantial global burden, only one vaccine is licensed (in China and Pakistan), limiting its global accessibility. Furthermore, no globally approved mRNA vaccine against HEV is currently available, highlighting the need to develop strategies for alternative vaccines.

**Methods:**

In this study, the amino acid sequences of ORF2 capsid proteins representative of HEV genotypes 1–4 were curated, retrieved, and analyzed to generate a consensus sequence. The conserved cytotoxic T lymphocyte (CTL), helper T lymphocyte (HTL), and B-cell epitopes were computationally screened based on their antigenicity, allergenicity, toxicity, IFN-γ induction, and human non-homology. Then, the selected epitopes, seven in total, were assembled into a computational multi-epitope mRNA construct incorporated with β-defensin adjuvant, correct linkers, and regulatory elements [Kozak, tPA, MITD, and globin untranslated regions (UTRs)]. The construct was subsequently subjected to structural modeling, refinement and validation, docked with the TLR1/TLR2 heterodimer, followed by normal mode analysis and a 100-ns molecular dynamics simulation with MM-GBSA binding free-energy estimation, immune simulations, codon optimization, and *in silico* vaccine construct design.

**Results:**

The computational analyses predicted that the proposed epitopes possessed a favorable antigenicity (VaxiJen score ≥0.4–1), with non-allergenicity and epitope conservancy (80%–100%) across the analyzed HEV genotypes (1–4), and that the assembled vaccine construct was structurally stable. Protein–protein docking of the vaccine construct candidate with the TLR1/TLR2 heterodimer and normal mode analysis of the docked complex indicated a stable structural interface rather than a confirmation of biological receptor engagement; a subsequent 100-ns molecular dynamics simulation supported a persistent, increasingly hydrogen-bonded interface with a favorable MM-GBSA binding free-energy (−91.96 kcal/mol). Computational immune simulations predicted cellular and humoral immune responses with B- and T-cell interaction activity and cytokine production levels.

**Conclusion:**

This study describes a computationally designed multi-epitope mRNA vaccine candidate with predicted immunogenicity and structural stability against the major human-infecting HEV genotypes 1–4. These computational results support the proposed vaccine construct as a promising candidate for further research to assess its immunogenicity; safety and protective efficacy will require experimental validation before its translational potential is verified.

## 1 Introduction

Hepatitis E virus (HEV) is a virus transmitted through fecal–oral contamination that causes inflammation of the liver, known as acute hepatitis, in humans. Globally, it is estimated that HEV-induced acute hepatitis affects approximately 20 million people annually (Lynch et al., 2023). The virus is common in Asia, Africa, and the Middle East (particularly prevalent in developing countries), where it is classified as endemic, although sporadic cases have been reported globally (Zahmanova et al., 2023; Bienz et al., 2024). The infection is self-limited and can typically resolve without requiring hospitalization; however, it poses a substantial risk with potentially severe repercussions for immunocompromised individuals such as individuals with AIDS, pregnant women, and those suffering from chronic liver disease (Chaudhry et al., 2015). Genetically, HEV can be defined as a positive-stranded RNA virus with eight distinct recognized genotypes that infect humans and several animal species including pigs, wild boars, and camelids (Melchert et al., 2023). The vast majority of confirmed human infections are of genotypes 1–4. Genotypes 1 and 2 are transmitted predominantly through contaminated water and are highly associated with large outbreaks in developing regions, whereas genotypes 3 and 4 are zoonotic and are commonly acquired through the consumption of undercooked meat or through direct contact with infected animals (Okamoto et al., 2003). These genotypes are associated with different geographical regions and have distinctive characteristics. Genotype 1, also known as the Burmese strain, is the most prevalent type in Asia and Africa. Genotype 2, known as the Mexican strain, is endemic in Mexico, Nigeria, and Chad. Genotype 3 has a global distribution and is found in both developed and developing countries. Finally, genotype 4, known as the Chinese strain, is found in Asia (Smith et al., 2013). This study, consequently, focused on HEV genotypes 1–4 as they constitute the major human-infecting genotypes with the greatest global public health relevance and represent the principal targets for vaccine development.

HEV genotypes 5–8 have been characterized more recently and largely remain restricted to animal reservoirs, with only sporadic or unconfirmed human infection reported to date (Songtanin et al., 2023). They are outside the scope of the present study.

Structurally, the HEV virion is an icosahedral-shaped non-enveloped sphere ranging 27 nm–34 nm. The genome of HEV contains three major open reading frames: ORF1, ORF2, and ORF3. ORF1 of the HEV genome encodes a putative polyprotein, ORF2 encodes the capsid protein, and ORF3 codes for VP13. These proteins and ORFs are the most important focus of the HEV vaccine development (Lu et al., 2024). Among these three, ORF2 is the principal structural antigen and the primary target of neutralizing antibodies owing to its strong immunogenicity and relatively high sequence conservation. ORF2 has been analyzed extensively as a vaccine antigen in both computational and experimental studies; thus, it was selected as the target antigen for the present immunoinformatics-based vaccine design (Ikram et al., 2023).

The introduction of nucleic acid vaccines has revolutionized the concept of disease prevention (Rehman et al., 2023) as these platforms have a clear advantage over conventional recombinant vaccines, propelling increasingly complex strategies such as mRNA vaccine design. Recently, the use of mRNA vaccines in the COVID-19 pandemic highlighted their role as a safe and cheaper substitute for standard vaccine production technologies (Gote et al., 2023; Riaz et al., 2024). Unlike conventional vaccine platforms, mRNA vaccines permit the incorporation of multiple conserved epitopes into a single construct while eliminating the need to culture infectious organisms (Lokras et al., 2024). These characteristics make mRNA technology particularly well-suited for immunoinformatics-guided vaccine design against genetically diverse viral pathogens such as HEV (Chaudhary et al., 2021). The mRNA vaccinology’s success rate has transformed modern vaccine technology by enabling rapid antigen design, scalability to manufacture, and flexibility to adapt to emerging pathogens (Leong et al., 2025).

Furthermore, the current situation is exacerbated by the limitation that only one vaccine (HEV 239) is available, and its use is restricted to China and Pakistan. It is not yet authorized worldwide by WHO prequalification (Zahmanova et al., 2023). These factors render it essential to develop an effective HEV vaccine for use in protecting against all genotypes of HEV infecting humans, which is not only effective but also producible in a cost-effective manner. Consequently, there remains a need to explore alternative vaccine platforms that can be rationally designed to target the major human-infecting HEV genotypes. In this context, immunoinformatics-guided mRNA vaccine design provides a flexible computational framework for the identification and integration of conserved immunogenic epitopes into a single vaccine construct (Wei et al., 2024; Suneesh et al., 2025).

The HEV 239 vaccine is derived from the ORF2 capsid protein of a single HEV genotype 1 and is described as a recombinant subunit vaccine. It is expected to confer cross-protection against genotypes 2–4 based on shared antigenic determinants, although this has not been established through genotype-specific clinical efficacy data (Peron et al., 2023). Thus, a multi-epitope mRNA-based approach allows for an explicit incorporation of curated epitope candidates for conservancy from genotypes 1–4 at the designing stage level rather than relying on untested cross-reactivity from a genotype 1-based derived antigen (Marcos-Villar et al., 2026).

There are several computational vaccine designs targeting HEV that have been reported, but many focused on individual viral proteins (Ikram et al., 2023). They are either limited to or restricted to genotype representation (Lu et al., 2024; Ikram et al., 2023) or have not applied a multi-parameter screening pipeline across all four major human-infecting genotypes 1–4, integrating antigenicity, allergenicity, toxicity, human proteome homology, and per-genotype conservancy at the individual epitope level. A full feature comparison against these three prior HEV multi-epitope designs is provided in Supplementary Data. Accordingly, the present study describes the computational design and *in silico* evaluation of a multi-epitope mRNA vaccine candidate targeting the four major human-infecting HEV genotypes (1–4) using an integrated immunoinformatics workflow.

## 2 Methodology

### 2.1 Protein sequence retrieval

The NCBI protein database was used to retrieve protein sequences of structural proteins that are encoded by HEV ORF2 (Sayers et al., 2026). This retrieval was inclusive of all prominent human-infecting genotypes of the hepatitis E virus genotypes 1–4. A 600–680 amino acid range was filtered to retain complete or near-complete ORF2 proteins. The selected sequences from human isolates assigned to genotypes 1–4 were retained, and the sequences with unclear records of genotype annotation, non-human hosts, incomplete sequences, or duplicate accessions were excluded (Supplementary Data). The manually reviewed sequences from UniProt representative reference sequences were only eligible and included after duplicate checking. The sequences were imported into UGENE (Okonechnikov et al., 2012), and MUSCLE (Edgar, 2004) was used for multi-alignment. From this approach, a consensus of the sequences was generated for the downstream prediction and conservancy assessment of epitopes.

### 2.2 Prediction of T-cell and B-cell epitopes and feature profiling of the selected epitope candidates

The servers NetMHCpan-4.1 and NetMHCII-2.3 were used for the prediction of MHC class I and MHC class II epitopes, respectively, run in eluted ligand (EL) mode (Reynisson et al., 2020). The standard IEDB-recommended threshold for EL-mode predictions was set at %rank <0.5 to predict peptides and defined as strong binders (SB). The prediction of epitopes for MHC class I allele sets [HLA-A and HLA-B ] and MHC class II allele sets [HLA-DP, HLA-DQ, and HLA-DR] was generated separately, and candidate peptides appearing as exact-sequence duplicates across multiple alleles were collapsed to yield a unique peptide set per class. Both MHC class I and MHC class II candidate pools were then passed through the same sequential mandatory filters: antigenicity (VaxiJen ≥0.40) (Doytchinova and Flower, 2007), allergenicity (AlgPred2 threshold ≤0.30) (Sharma et al., 2021), toxicity (ToxinPred3 ≤ 0.38) (Rathore et al., 2024), and PEPMatch human proteome exclusion (0 mismatches for exact matches and 1 mismatch for near matches) (Marrama et al., 2023). MHC binding is determined by the short cores, and post filtration, all of the epitopes were subjected to a 7mer, 8mer, and 9mer sliding window based the PEPmatch assessment. This led to 96 unique queries, which were searched against the human proteome, including isoforms and un-reviewed entries, allowing up to three substitutions. All exact and single-substitution matches were reassessed for binding to the HLA allele originally associated with the corresponding epitope, using NetMHCpan 4.1 (class I) and NetMHCII 2.3 (class II, the original selection model). Human peptides were reconstructed with native flanking residues to 9-mers (class I) or 15-mers (class II) and compared with the viral peptides on predicted binding-core overlap and binding register. Non-gating characterization was then applied across the full candidate pool: IFN-γ induction (IFNepitope2 threshold ≤0.49) (Dhall et al., 2024), exact-match conservancy against eight reference sequences, and IEDB class-I immunogenicity 3.0 scoring (Calis et al., 2013) (validated interpretation restricted to 9-mers). The immunogenicity score was subsequently weighted in the final ranking and selection of MHC class-I candidates, while IFN-γ-induction evidence was weighted in the final ranking and selection of MHC class-II candidates. The final selected epitope candidates were mapped to their ORF2 structural domains (PDB 2ZTN): shell (S) domain, residues 129–319; middle (M) domain, residues 320–455; protruding (P) domain, residues 456–606 (Yamashita et al., 2009). Linear B-cell epitopes were predicted using ElliPro (Ponomarenko et al., 2008), BepiPred 2.0 (Je et al., 2017), and ABCPred (Saha and Raghava, 2006). Each candidate epitope was scored against 10 framework criteria, and seven were chosen as supporting prediction evidence criteria: ElliPro mean score ≥0.50, BepiPred 2.0 mean score ≥0.50, ABCPred best window ≥0.51, Karplus–Schulz mean flexibility ≥1.00 (Karplus and Schulz, 1985), DiscoTope 3.0 epitope residue proportion ≥50% (Høie et al., 2024), NetSurfP3.0 relative solvent accessibility ≥50% (Høie et al., 2022), and CABS-FLEX mean RMSF ≥1.00 Å, along with the mandatory filter mentioned above (Jamroz et al., 2013).

### 2.3 Design of vaccine construct

The predicted epitope candidates that passed all of the profiling features of T-cell and B-cell peptides were linked using linkers such as AAY [between CTL], GPGPG [between the HTL], and GGGGS [before the B cell] that are fused to an EAAAK helical linker connecting to an adjuvant β-defensin [Q5U7J2,45 aa residues] to the backbone of the vaccine-construct candidate.

### 2.4 Physicochemical and immunological properties

They were then assessed for their physicochemical properties using the ProtParam library. Their instability index, isoelectric point, aliphatic index, and GRAVY index were used to filter the constructs. Once selected, the sequences were also checked on ExPASy (Gasteiger et al., 2003) to retrieve a more detailed physicochemical analysis of the constructs.

### 2.5 Population coverage analysis

The IEDB population coverage tool (http://tools.iedb.org/population/) was used to estimate the proportion of the global population, with the regional and global population coverage estimates, including average hits and PC90 values, and to predict the response of the selected T-cell epitopes based on their associated HLA class-I and HLA class-II alleles. The analysis was conducted using the combined HLA class-I and class-II mode. The HLA alleles included in the analysis were those associated with the shortlisted MHC-I and MHC-II epitopes identified during T-cell epitope prediction.

### 2.6 Secondary and tertiary structure prediction and validation

The Vienna package version 2.0 was used to predict the secondary structure of the vaccine, which comprised α and β helices and coils (Lorenz et al., 2011). RNAfold was used to evaluate the secondary structure of the mRNA construct of the vaccine (Gruber et al., 2008). This program predicts the RNA centroid secondary structure and calculates the minimum free energy (MFE) structure using McCaskill’s algorithm. AlphaFold3 was used to predict the tertiary structure of the vaccine construct candidate (Abramson et al., 2024). AlphaFold3’s model confidence is assessed using the predicted local distance tests [mean and median pLDDT], predicted template modeling scores [pTM], predicted aligned error [PAE], and 90th percentile PAE. The final structure was sequentially refined by GalaxyRefine, GalaxyRefine2, and once more by GalaxyRefine, selected on the basis of their GDT-HA, RMSD, and MolProbity (Chen et al., 2010) scores. The final refined structure was assessed and validated using standard tools such as ERRAT (Colovos and Yeates, 1993) and Verify3D (Bowie et al., 1991).

### 2.7 Molecular docking

ClusPro 2.0 (Kozakov et al., 2017) was used for molecular docking of the final refined vaccine candidate construct core against Toll-like receptor TLR1/TLR2 heterodimer; its crystal structure (PDB 2Z7X) (Jin et al., 2007) was selected as the docking receptor on the basis that human β-defensin, the adjuvant incorporated into this construct, activates antigen-presenting cells through a mechanism requiring the expression of both TLR1 and TLR2 (Funderburg et al., 2007), consistent with the TLR1/TLR2 heterodimer as the physiologically relevant receptor target. ClusPro 2.0 generates 10 docking models per interaction. The best model with the lowest binding energy and the largest cluster size, which indicates the most stable receptor–ligand complex, was selected, and the balanced scoring scheme was applied; the top-ranked cluster’s center model was selected as the representative docked pose. PISA (Krissinel and Henrick, 2007) was used to assess the interface energetics.

### 2.8 Normal mode analysis

Normal mode analysis (NMA) of the docked complex was performed using iMODS (López-Blanco et al., 2014), which applies an elastic network model in internal coordinates to characterize potential conformational changes and assess complex stability. Structural flexibility was described through the main chain deformability plot, which measures the capacity of the structure to deform at each residue. Eigenvalues represent motion stiffness, which is directly related to the energy required to deform the structure; variance is shown as both individual and cumulative and is inversely related to the eigenvalue. The B-factor profile describes the magnitude of fluctuation at each residue, while the covariance map indicates whether pairs of residues move in a correlated, uncorrelated, or anti-correlated manner. The elastic network model represents the set of pairwise atomic interactions underlying these calculated motions.

### 2.9 Molecular dynamics simulation

The docked TLR1/TLR2 vaccine complex was subjected to explicit solvent MD simulation to assess the complex’s stability beyond the coarse elastic network approximation of NMA. Structural parameters were generated using the AMBER99SB-ILDN force field (Lindorff-Larsen et al., 2010). The complex was solvated in a TIP3P triclinic water box with a minimum 1.0-nm solute-to-edge distance, and 9 Cl- counterions were added for charge neutrality. Energy minimization was performed using the steepest-descent algorithm. A convergence criterion of 10 kJ mol^-1^ nm^-1^ and a maximum of 50,000 steps was used, which was followed by 100 ps each of NVT and NPT equilibration, before a 100-ns production simulation using the leap-frog integrator (GROMACS v2026.2) (Abraham et al., 2015). RMSD (receptor backbone, vaccine construct backbone, and vaccine construct backbone after alignment to the receptor), RMSF, radius of gyration, solvent accessible surface area, and intermolecular hydrogen bond count were calculated over the trajectory. A free energy landscape was constructed by projecting Cα atom motion onto the first two principal components. Binding free-energy was estimated by MM-GBSA on 100 equidistant frames using gmx_MMPBSA (Valdés-Tresanco et al., 2021). Protein–ligand interactions in the final trajectory frame were visualized with LigPlot+ v2.3.1 (Laskowski and Swindells, 2011).

### 2.10 Immune stimulation

To examine the immunological response evoked by the finalized chimeric vaccine construct, *in silico* immune simulation analysis was performed using the C-IMMSIM server (Rapin et al., 2010). This server operates by using a position-specific score matrix [PSSM] and machine learning techniques to predict the host’s immune response toward multi-epitope vaccine constructs. Vaccine construct immune simulation was conducted according to a previously used method (Kakakhel et al., 2023). The input criteria for the simulation parameters were kept at default values, i.e., random seed = 12,345 and volume of the simulation = 10, and the vaccine injection did not contain LPS. A single injection of 1,000 antigen molecules (adjuvant = 100) was administered at time step 1. The immune simulation was conducted for 100 simulation steps, along with human host leukocyte antigen selection homozygous for [HLA-A01:01, HLA-B07:02, and HLA-DRB1*01:01].

### 2.11 Codon optimization and *in silico* vaccine construct design

BackTranSeq (https://www.ebi.ac.uk/Tools/st/embossbacktranseq/) was used to reverse translate the vaccine construct to obtain the nucleotide sequence. The JCat tool (Grote et al., 2005) optimized the codons for the host organism [*Homo sapiens*]. ViennaRNA and NetGene2 2.4 [*Homo sapiens* model] (Hebsgaard et al., 1996) were used to assess the optimized JCat sequence subsequently for the GC content distribution, dinucleotide frequency, secondary structure accessibility at the start codon, cryptic splice site potential, and exact sequence repeats. These analyses identified strong secondary structure predicted to interfere with ribosomal access at the start codon; synonymous targeted substitutions were introduced to make the start codon accessible and reduce the cryptic sites. The GC content was separately calculated for the coding region positions from 80 to 784, the non-poly (A) transcript positions from 1 to 915, and the full transcript positions, which included the poly (A) tract positions from 1 to 1,035 since the whole GC content is lowered by the poly(A) by design and is not representative of the coding region compositions. The overlapping adjacent pairs were calculated as f(XY)/f[(X).f(Y)] of the expected or the observed ratios of CpG and UpA dinucleotides.

SnapGene software (www.snapgene.com) was used for *in silico* vector map construction and cassette design. The pIVTRup plasmid (RRID: Addgene #101362) for the backbone IVT was selected/chosen for its T7 promoter, and it established the architecture to generate a linear DNA template for mRNA synthesis. The complete optimized 1,035 bp mRNA cassette; 5′ UTR, Kozak sequence, tPA signal peptide, β-defensin adjuvant, EAAAK linker, multi-epitope sequence, MITD sequence, triple stop codons (UAA, UGA, and UAG), 3′UTR, and a 120 nucleotide poly(A) tail replaced the native pIVTRup transcript cassette for *in silico* cloning. The cassette replacement was used instead of simple insertion to avoid the duplication of vector-derived UTR and poly (A) sequence. In the correct transcriptional orientation, a single T7 promoter is retained upstream of the cassette. For the proposed IVT, the complete transcript unit was designed to be amplified from the recombinant plasmid by PCR (high fidelity). The incorporation of a 5′ m^7^GCap structure was proposed utilizing a suitable capping system following transcription. Thus, no experimental cloning, PCR, *in vitro* transcription, or capping was performed.

## 3 Results

### 3.1 Identification and sequence retrieval from the conserved regions in HEV ORF2

The curation process yielded a final dataset of 440 HEV ORF2 sequences: genotype 1, 23; genotype 2, 3; genotype 3, 319; and genotype 4, 95. Genotype 2’s comparatively low representation reflects the limited public availability of molecular record and the restricted surveillance coverage for that genotype (Wang et al., 2018) rather than an additional exclusion criterion. Full inclusion/exclusion criteria, final composition by genotype, and geographic distribution are provided in Supplementary Data. The conservation analysis was utilized to construct a multi-epitope HEV mRNA vaccine that encompassed the prevalent genotypes. The total length of the conserved sequence was found to be 660 amino acids.

### 3.2 Prediction and feature profiling of T-cell and B-cell epitopes

The servers NetMHCpan-4.1 and NetMHCII-2.3 were run in the EL mode and predicted 66,156 total parsed rows across MHC class-I and MHC class-II allele sets [31,272 (HLA-A/B), 16,150 (HLA-DR), 5,814 (HLA-DP), and 12,920 (HLA-DQ), respectively]. Strong binding was set at % rank <0.5, which retained 1,127 hits. These were further narrowed to 481 unique peptide sequences after being excluded due to duplication by exact amino acid sequence hits across the records and passed through the sequential mandatory filters. This further reduced the pool to 66 candidate epitopes (Supplementary Data; see Supplementary Flowchart Figure and Supplementary Table). The non-gating characterization of the 66 candidate epitopes identified 17 of them as IFN-γ inducers, and seven were independently identified as meeting the IEDB immunogenicity eligibility criteria; 23 peptides showed 100% exact-reference conservancy. Immunogenicity score was subsequently weighted in the final ranking of MHC class-I candidates, while IFN-γ induction evidence was weighed in the final ranking of MHC class-II candidates. These epitopes were then selected and prioritized for the final vaccine construct candidate, consisting of four MHC class-I and two MHC class-II epitopes. All four finalized MHC class-I epitopes were HLA-B allele-restricted. The ORF2 structural domain assignment of these epitopes is reported in Supplementary Data. A total of 99 unique linear B-cell candidate epitopes were identified across ElliPro (13), BepiPred 2.0 (22), and ABCPred (64). A total of 16 candidate epitopes were retained after the application of the mandatory sequential filters described in Methods (Supplementary Data). These were further evaluated against the seven structural and biophysical evidence criteria. One B-cell epitope was selected by passing six out of the seven evidence criteria at position 479–493. It showed 80% exact reference conservancy and was reported to lie within both the ORF2 neutralizing region and the protruding [P] structural domain (Supplementary Data). One additional candidate was deprioritized after failing three of the seven evidence criteria and showing no neutralizing overlap (Supplementary Data). A screening yielded 48,582 non-redundant matches, of which 118 showed one or fewer substitutions. On same-HLA reassessment, 36 human peptides across 27 genes retained binding to their corresponding viral epitope’s HLA allele, nine of which bound at least as strongly as the viral epitope itself; among these nine, shared residues with the corresponding viral epitope ranged from 7 out of 9 (the sole MHC class-I match, ABCB8) to 6–8 out of 15 (each of the eight MHC class-II matches). One of the 118 near-exact events was the one-mismatch match between the MHC class-I epitope GEVGRGIALTL and a human ABCB8-derived peptide, which retained the predicted HLA-B*40:01 binding (Supplementary Data). No similarity anywhere in the dataset exceeded eight shared residues, which was below the nine residues of a complete MHC class-II binding core. Separately, exact-match screening identified no 9-mer or 8-mer match; one exact 7-mer match was found within the MHC class-II epitope SVLRANDVLWLSLTA (human apelin), a non-binder for two of three associated HLA-II alleles and a weak binder for the third. Per-genotype conservancy was additionally assessed against reference ORF2 sequences for genotypes 1–4 individually (Supplementary Data). Five of seven epitopes showed 100% conservancy across all four genotypes. Two epitopes showed genotype-1-specific variation: GEVGRGIALTL at 90.91% (a single V3I substitution present in all five genotype 1 references) and KTFYVLPLRGKLSFW at 86.67%–93.33% depending on the reference. EYDQTTYGSSTNPMY, the B-cell epitope, showed 80% conservancy in genotypes 1 and 2 and 100% conservancy in genotypes 3 and 4, indicating that the residual sequence variation in the finalized construct is concentrated in genotypes 1 and 2 rather than distributed evenly across the four target genotypes. AIPpred was used as an additional tool to characterize all seven selected epitopes in the finalized construct for anti-inflammatory potential; all were classified as anti-inflammatory epitopes (AIP) at probabilities ranging from 0.3849 to 0.4442 (Supplementary Data), which is close to the classification threshold. This is reported as secondary characterization rather than a selection criterion; no epitope was excluded or reweighed on this basis.

### 3.3 Design of vaccine construct

The final vaccine construct comprised the following sequential elements from the N-terminal to the C-terminal: 5′ m7GCap, 5′ UTR, Kozak sequence, signal peptide (tPA), adjuvant, EAAAK linker, multi-epitope vaccine construct sequence, MITD sequence, stop codon, 3′ UTR, and poly (A) tail (Figure 1). This combination of vaccine construct showed favorable antigenicity and was selected as the vaccine candidate construct. The complete translated construct, comprising the tPA signal peptide, β-defensin adjuvant, EAAAK linker, epitope core, and MITD domain, contains 235 amino acids; the 158-residue adjuvant–linker–epitope core described in physicochemical and structural analyses below excludes the cleavable tPA and MITD trafficking elements.

**FIGURE 1:**
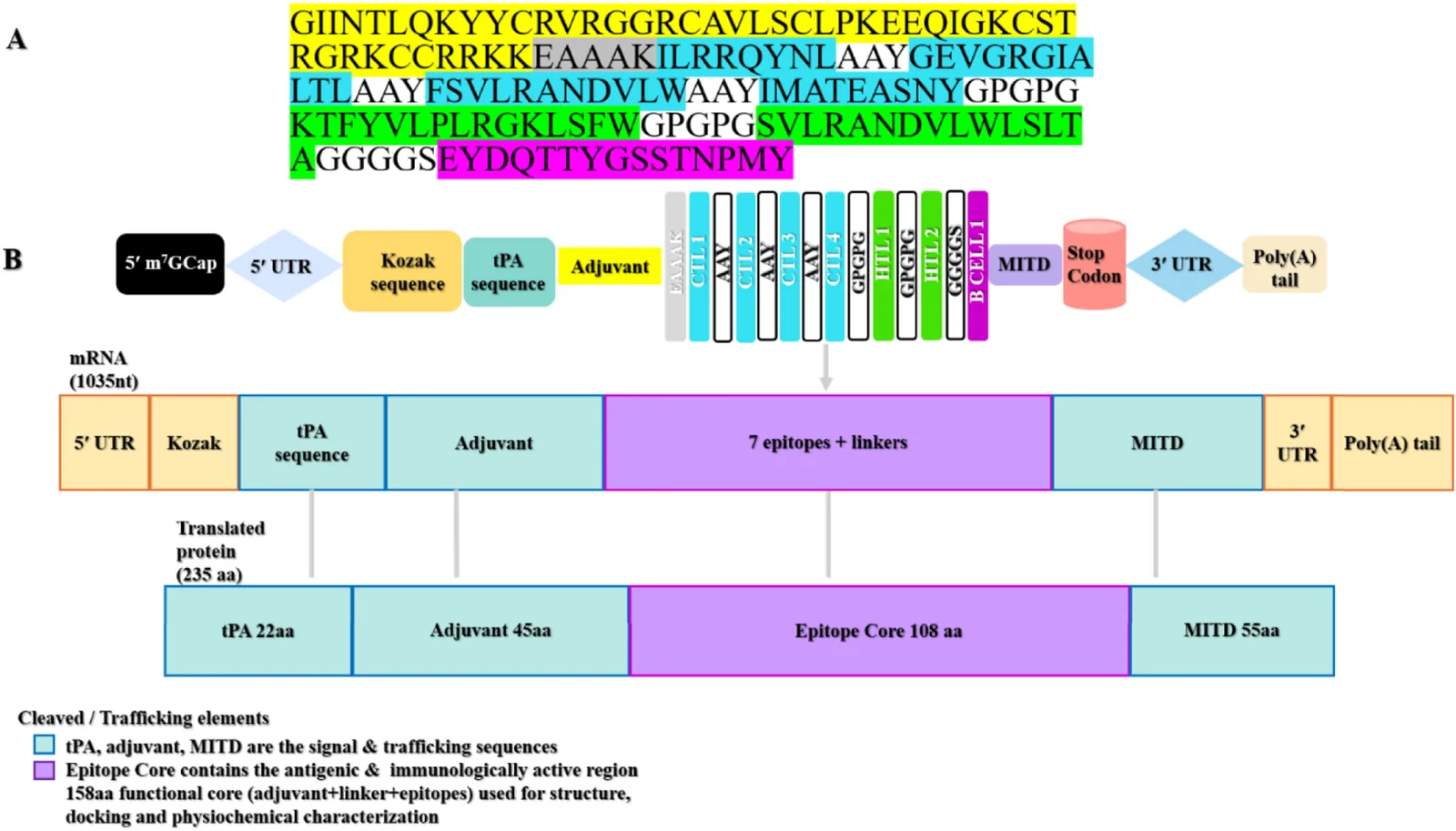
**(A)** Schematic representation of the vaccine construct showing the amino acid sequence organization and color-coded components. The β-defensin adjuvant is shown in yellow, the EAAAK linker is shown in gray, cytotoxic T lymphocyte (CTL; MHC class I) epitopes are shown in cyan, helper T lymphocyte (HTL; MHC class II) epitopes are shown in green, and the linear B-cell epitope is shown in magenta. **(B)** Structural organization of the complete mRNA vaccine construct illustrating the 5′ m7G cap, 5′ untranslated region (UTR), Kozak sequence, tissue plasminogen activator (tPA) signal peptide, β-defensin adjuvant, multi-epitope region joined by appropriate linkers, MHC class-I trafficking domain (MITD), stop codon, 3′ UTR, and poly(A) tail. The lower panel shows the corresponding translated protein architecture, highlighting the tPA signal peptide (22 aa), β-defensin adjuvant (45 aa), multi-epitope core (108 aa), and MITD sequence (55 aa). The translated vaccine construct (158 aa) was used for subsequent physicochemical characterization, structural modeling, and molecular docking.

### 3.4 Population coverage of epitopes analysis across South Asia and worldwide

Population coverage was calculated using the IEDB Population Coverage tool (available at https://tools.iedb.org/population/) (class I and class II combined mode) against the finalized allele set HLA-B*15:01, HLA-B*40:01, HLA-B*58:01, and HLA-B*08:01 for the MHC I epitopes and HLA-DQA1*03:03, HLA-DQA1*05:01, HLA-DQA1*06:01, HLA-DQB1*04:02, HLA-DRB1*01:01, HLA-DRB1*08:01, HLA-DRB1*08:02, HLA-DRB1*11:01, HLA-DRB1*04:03, and HLA-DRB1*13:02 for the MHC II epitopes (Table 1); HLA-DRB3*03:01 was identified in the epitope-binding predictions but was not recognized by the IEDB Population Coverage tool and was, therefore, excluded from the coverage calculation. Global coverage was 75.87%, and South Asian coverage was 62.82% (Figure 2). Coverage was additionally calculated across 16 continental and HEV endemic regions (Supplementary Data), ranging from 22.16% (South Africa) to 88.30% (Central America), with a 16-region average of 71.27% (SD 14.45). Coverage was notably lower in South Africa (22.16%) and Southwest Asia (60.84%) relative to other regions, which we attribute to the absence of HLA-A and HLA-C representation among the finalized MHC I epitopes (all four are HLA-B restricted); this is discussed further as a limitation.

**TABLE 1:** Detailed list of the predicted, identified, and selected MHC class I, class II, and B-cell epitopes and their parameters.

Epitope type	Selected peptide	Length (aa)	Position (ORF2)	Binding (%rank) score	HLA alleles	Antigenicity	Allergenicity	Toxicity	Conservancy per genotype	IFN-γ induction	Human non-homolog
MHC I	IMATEASNY	9	174–182	0.093% (EL = 0.8247)	HLA-B*15:01	Antigen 0.7715	Non-allergenic	Non-toxic	100%	Inducer	No exact match (0 mismatch)
	GEVGRGIALTL	11	362–372	0.124% (EL = 0.8589)	HLA-B*40:01	Antigen 0.9305	Non-allergenic	Non-toxic	90.91%–100%	Inducer	One-mismatch match (human ABCB8-derived peptide; retains HLA-B*40:01 binding)
	FSVLRANDVLW	11	462–472	0.179% (EL = 0.7938)	HLA-B*58:01	Antigen 0.7583	Non-allergenic	Non-toxic	100%	Non-inducer	No exact match (0 mismatch)
	ILRRQYNL	8	131–138	0.240% (EL = 0.4817)	HLA-B*08:01	Antigen 1.0127	Non-allergenic	Non-toxic	100%	Non-inducer	No exact match (0 mismatch); no
MHC II	KTFYVLPLRGKLSFW	15	534–548	0.5% (affinity = 0.8554)	HLA-DQA1*03:03, HLA-DQA1*05:01, HLA-DQA1*06:01, HLA-DQB1*04:02, HLA-DRB1*01:01, HLA-DRB1*08:01, HLA-DRB1*08:02, and HLA-DRB1*11:01	Antigen 0.9170	Non-allergenic	Non-toxic	86.67%–100%	Inducer	No exact match (0 mismatch)
	SVLRANDVLWLSLTA	15	463–477	0.7% (affinity = 0.8488)	HLA-DRB1*04:03, HLA-DRB1*13:02, and HLA-DRB3*03:01	Antigen 0.9693	Non-allergenic	Non-toxic	100%	Inducer	Exact 7-mer match (human apelin)
B cells	EYDQTTYGSSTNPMY	15	479–493	-	-	Antigen 0.4871	Non-allergenic	Non-toxic	80%	Inducer	No exact match (0 mismatch)

**FIGURE 2:**
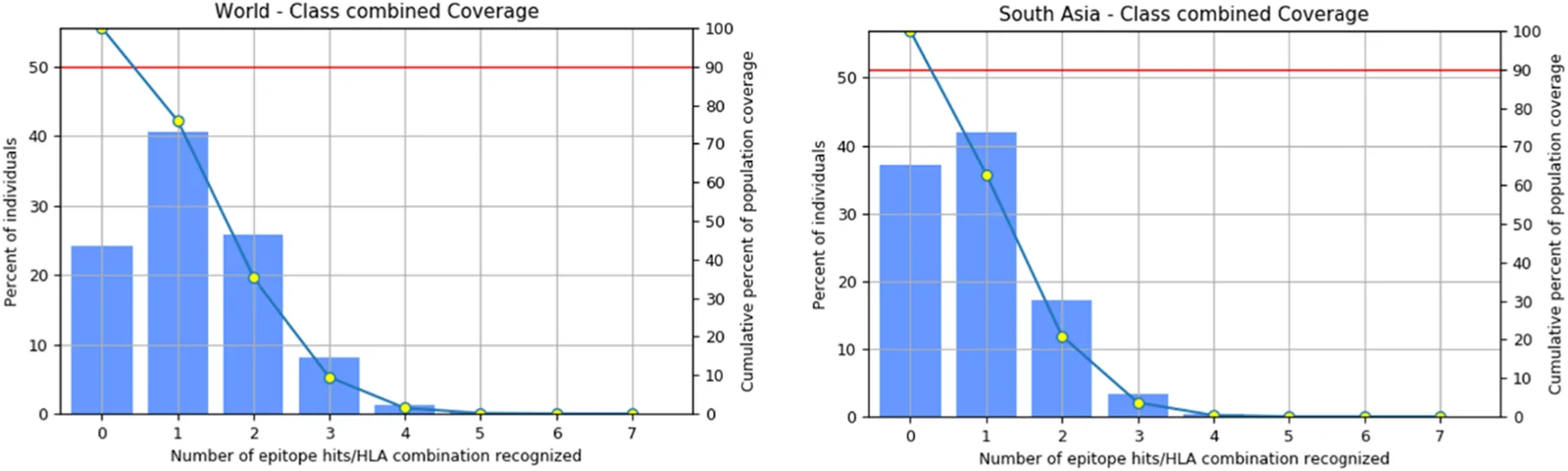
Population coverage analysis of combined coverage of the selected epitopes in the worldwide and South Asian population. The bars represent the population coverage for each epitope, and the line [-O-] represents the cumulative percentage of population.

### 3.5 Analysis of physicochemical properties prediction

ProtParam analysis of the construct revealed that the chemical structure of the construct comprised 235 amino acids and a molecular weight of 25004.02 Da (≈25 kDa). The theoretical pI was predicted to be 9.61, thus aiding in the design criteria. Additionally, the grand average hydropathy of the construct was 0.087, exhibiting thermal stability, and the instability index was 39.40, indicating that the structure is stable. The estimated half-life varies across systems, and the remaining properties are summarized in Supplementary Data.

### 3.6 Secondary and tertiary structure prediction and validation

The 2D structure of the vaccine construct was predicted using PSIPRED (McGuffin et al., 2000). These findings indicate that the composition of the vaccine’s structure consists of α-helices, β-sheets, and coils. The vaccine construct epitopes contain 158 amino acids. Of these, 49 [31.01%] amino acids participate in the formation of an α-helix, 36 [22.78%] form a β-strand, and 73 [46.20%] amino acids form a coil (Figure 3). The RNAfold server was utilized to predict the secondary structure of the mRNA vaccine model. The predicted models of the structural configuration of the mRNA displayed stability that was defined by a MFE metric of −235.36 kcal/mol for the mRNA vaccine construct in a characterized thermodynamic ensemble. The secondary structure centroids for the mRNA vaccine structure displayed a MFE of −194.70 kcal/mol and appeared in dot bracket notation, as shown in Figure 4. For the 3D structure prediction by AlphaFold3 of the final vaccine construct (Figure 5), the complete amino acid candidate construct was predicted and selected. The models generated were ranked based on the threshold provided by the server ranking scores and the top-ranked scores. Model 0 was selected for refinement. The model showed a mean pLDDT of 54.3 (median 43.81) and a pTM score of 0.31, with a mean predicted aligned error (PAE) of 21.61 Å and a 90th percentile PAE of 30 Å. The structure was sequentially refined by GalaxyRefine, GalaxyRefine2, and once again by GalaxyRefine. Model 5 of Model 0 was selected on the basis of values of GDT-HA 0.9541, RMSD 0.408 Å, MolProbity score 1.414, clash score 5.6, 0.0% poor rotamers, and 97.4% Ramachandran favored residues (Figure 6). The overall factor of 93.1034 ERRAT was achieved by the final structure and further assessed by a Verify3D score of 75.32% and validated by MolProbity analysis, which identified no poor rotamers and 100% favored rotamers, while the favored Ramachandran regions occupied 97.44% of residues and only 1.28% were outliers (two residues). The Z score of Ramachandran distribution was −0.76 ± 0.54.

**FIGURE 3:**
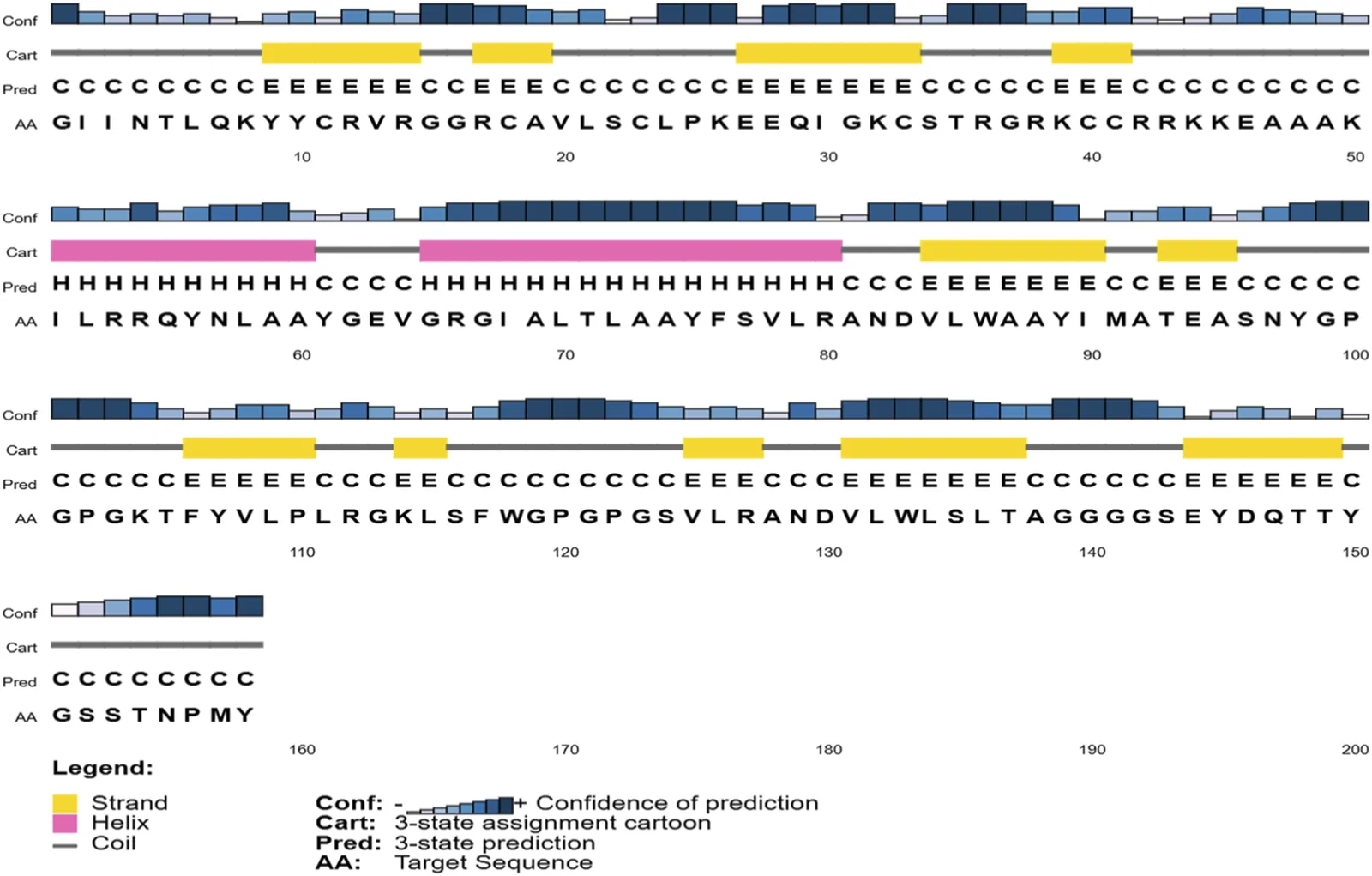
2D structure of the proposed vaccine construct, α-helices, β-strands, and random coils, which are represented in pink, yellow, and blue, respectively.

**FIGURE 4:**
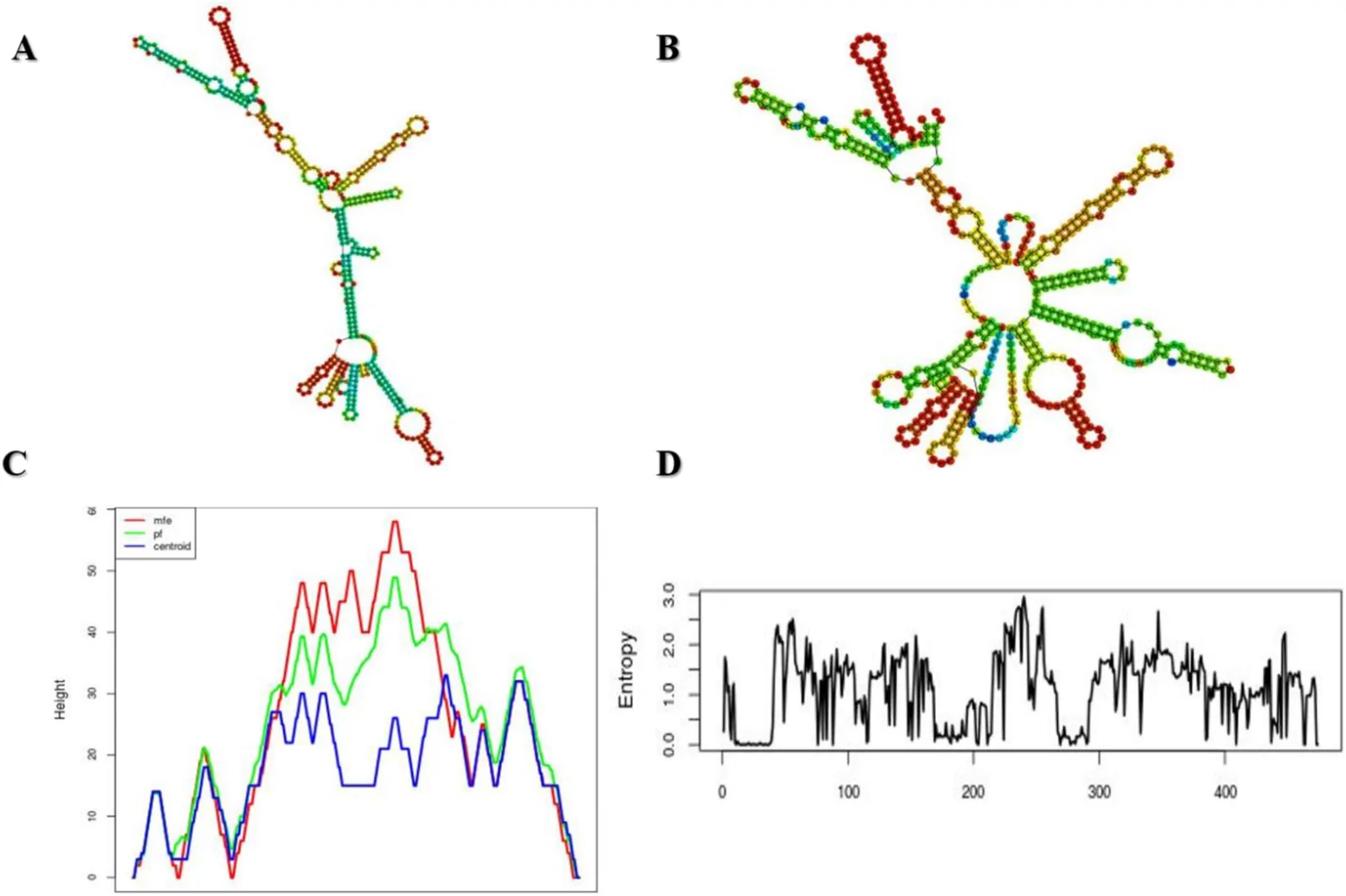
2D structure prediction of the prioritized mRNA vaccine construct. **(A)** Model presentation of minimum free energy of mRNA vaccine structure. **(B)** Proposed model of the minimum free energy of the secondary centroid mRNA vaccine structure. **(C)** Mountain plot representation of the MFE, thermodynamic ensemble of mRNA structure, and the centroid structure. **(D)** Positional entropy plot for each position.

**FIGURE 5:**
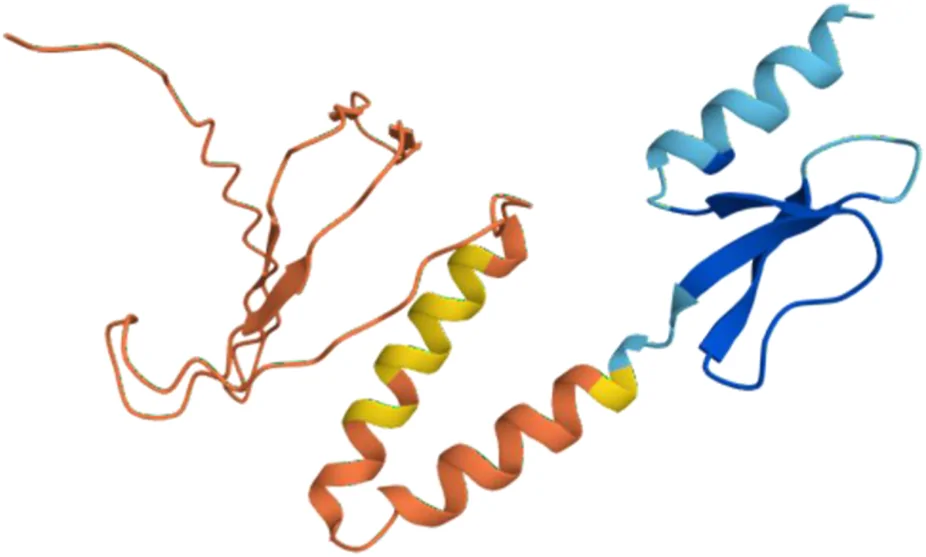
3D structure of the proposed vaccine construct, colored in rainbow from blue [N-terminus] to orange [C-terminus].

**FIGURE 6:**
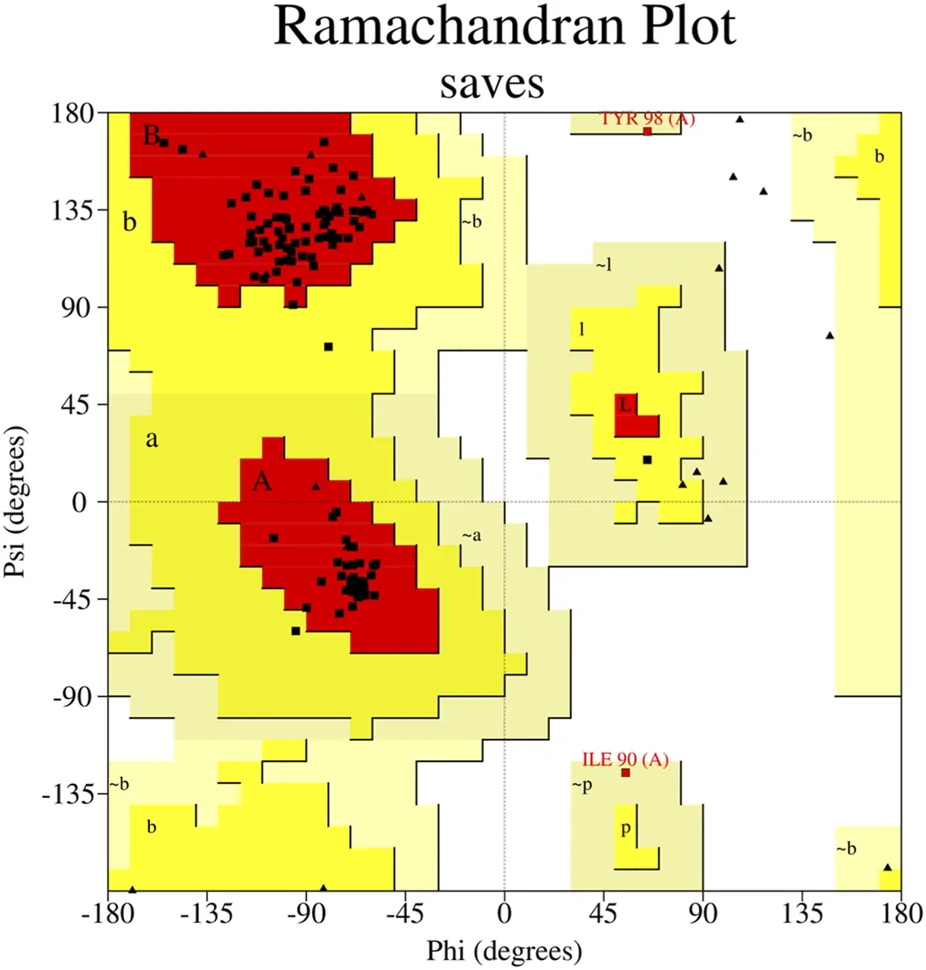
Ramachandran plot examination of the proposed vaccine construct by MolProbity. The plot shows that 97.4% of the residues are located in the most favored regions, with no residues in the disallowed regions.

### 3.7 Molecular docking

The vaccine construct core was docked against the TLR1/TLR2 heterodimer (PDB 2Z7X). ClusPro 2.0 identified a top cluster of 76 members under the balanced scoring scheme (center score −947.2, lowest energy score −1,067.6); the center model of cluster 0 was selected as the representative docked pose (Figure 7). PDBePISA interface analysis of the docked complex revealed an interface area of 935.0 Å^2^, involving 26 receptor residues and 25 vaccine construct residues (51 residues in total). The predicted interface solvation energy change (ΔiG) was −10.2 kcal/mol (*P* = 0.476); the non-significant *P*-value indicates that this interface is not statistically distinguished from typical crystallographic contacts by this metric alone. The interface was further characterized by nine hydrogen bonds and five salt bridge atom contacts corresponding to three unique salt bridge pairs; no intermolecular disulfide bonds were present.

**FIGURE 7:**
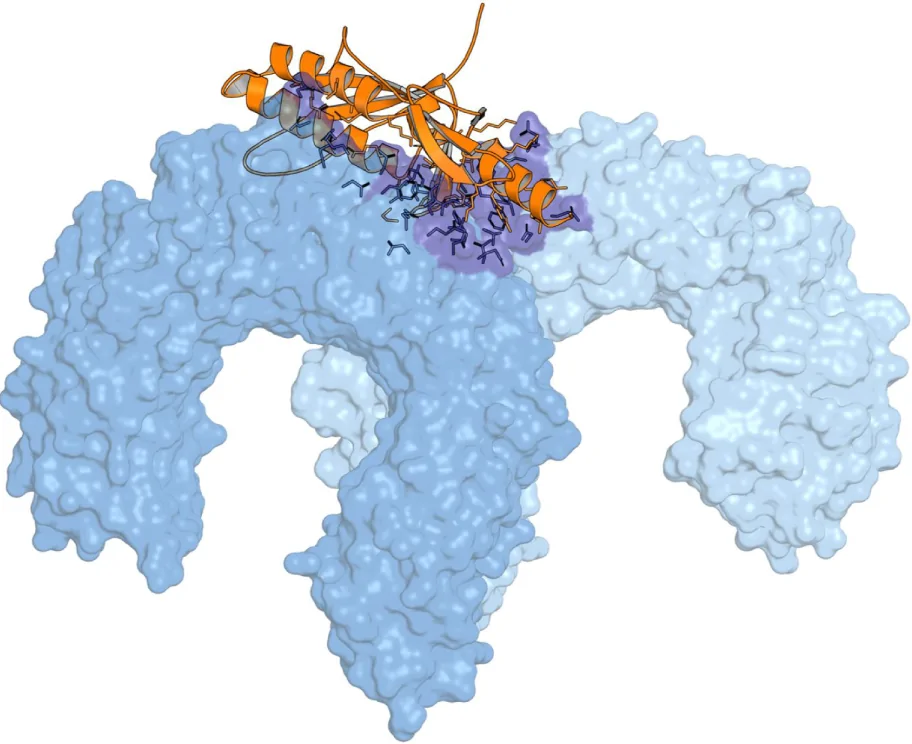
ClusPro-generated docked model (cluster 0, balanced scoring scheme, center model) of the vaccine construct core bound to the TLR1/TLR2 heterodimer (PDB 2Z7X); its ectodomain horseshoe is shown in salmon, with the β-strand and leucine-rich repeat regions highlighted in magenta and cyan; the core vaccine construct is shown in green, yellow, and red, positioned at the concave face of the receptor horseshoe.

### 3.8 Normal mode analysis

The first non-zero eigenvalue obtained using normal mode analysis was 1.174093 × 10^−5^ for the TLR1/2 construct complex using the iMODS elastic network model (Figure 8C). Both deformability and B-factor maps indicated that there is an increased prediction of flexibility in the loop regions of the complex (Figures 8A,B), which is in line with the observation made above on the disordered nature of the construct core. The variance analysis showed the first mode alone accounted for approximately 22% of the total motion, with cumulative variance exceeding 95% by mode 17 (Figure 8D). The covariance map also indicated both correlation and anti-correlation for collective motions in the complex (Figure 8E). The elastic network model showed core secondary structure elements as being comparatively stiff relative to the complex’s more flexible loop and terminal regions (Figure 8F). The results generated by normal mode analysis from iMODS are only considered rough predictions of the chosen docked structure and not as any proof of biological stability of receptor engagement.

**FIGURE 8:**
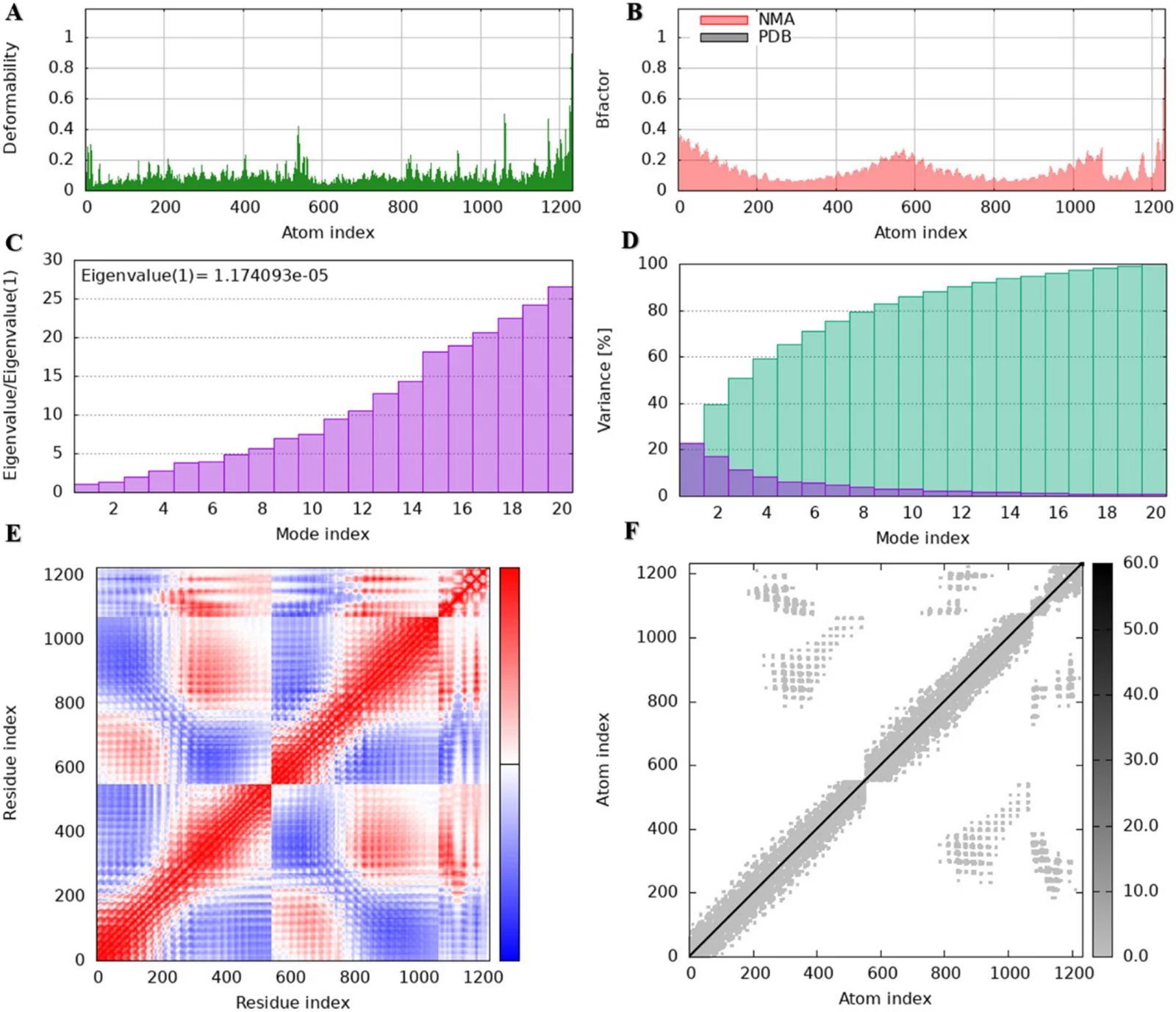
Normal mode analysis results of HEV vaccine construct from iMODS server. **(A)** Deformability; **(B)** NMA mobility. **(C,D)** Eigenvalues: purple shows the individual, and green shows the cumulative variances **(E)**. Covariance map highlighting the motion of pairs, where red indicates correlated movement, white shows no correlation, and blue stands for anti-correlated motions. **(F)** Elastic network model showing the stiffer region in gray.

### 3.9 Molecular dynamics simulation

The RMSD of the vaccine around the protein indicates that the vaccine construct stabilizes around the protein at ∼70 ns and onward, with RMSD fluctuations of 0.70–1.20. The RMSD of the vaccine backbone indicates continuous rearrangement from 0.5 nm to 0.8 nm (Figure 9A). The RMSF of TLR1/2 shows that both chains A and B exhibit a minimal level of residue fluctuation, generally below 0.2 nm, with two localized exceptions: chain A shows a fluctuation of approximately 0.4 nm near residue 245, and chain B shows a fluctuation of approximately 0.6 nm near its C-terminal region (residue ∼545). In comparison, the vaccine construct chain displayed greater fluctuations in the C-terminal region (residues 139–158), peaking at 1.26 nm at residue 158, with a secondary maximum near residues 140–143 (Figure 9B). The SASA results reveal a gradual decrease in the solvent accessible surface area of the TLR1/2 vaccine complex from approximately ∼580 nm^2^ at the beginning of the simulations to nearly ∼545 nm^2^ at the end of the 100-ns trajectory (Figure 9C). This decrease indicates a slower shift to a more compact shape and less solvent exposure. The Rg of the TLR1/2 vaccine complex was observed within a range of approximately 3.75 nm–3.85 nm throughout the trajectory, with fluctuations becoming stable at approximately 60 ns (Figure 9D). The number of hydrogen bonds formed between TLR1/2 and the vaccine construct increased from 10 to 15 during the initial part of the simulations to approximately 15–18 bonds at the end of the simulation. This increase in bonds indicates strengthening and stabilization of polar interactions at the TLR1/2 vaccine construct interface (Figure 9E). The FEL graph indicates that the TLR1/2 vaccine construct complex samples several conformations with quite high free energies of 2 kJ/mol–4 kJ/mol, shown by light blue contours, before reaching a stable, low-energy conformation (<1 kJ/mol) represented with a dark-blue downward spike (Figure 9F). Furthermore, the favorable binding between the TLR1/2 and vaccine construct was supported by MM-GBSA binding free-energy analysis. MM/GBSA binding energy calculations reveal a binding energy of −91.96 ± 2.11 kcal/mol for the TLR1/2 vaccine construct complex, indicating a net favorable, electrostatically dominated but substantially desolvation-penalized interaction, typical of protein–protein interfaces of this size. LigPlot+ analysis of the final trajectory frame identified 20 persistent hydrogen bonds, involving 12 vaccine construct residues, distributed across both TLR1/2 chains. On TLR chain A, bonds were observed between vaccine construct residues Lys32 and Glu383 (3.12, 2.79 Å), Arg42 and Asp327 (2.79, 2.90 Å), Gln29 and the His358/Gln357 pair (2.83, 3.10 Å), Lys50 and Asn300 (2.72 Å), Arg53 and Glu264 (2.79, 2.78 Å), and Glu46 and the Ser298/Ala297 pair (2.61, 2.72 Å) (Figure 9G). On TLR chain B, bonds were observed between Gly121 and Glu366 (2.98, 2.88 Å), Ser116 and Glu321 (2.92 Å), Trp118 and Glu321 (2.87 Å), Lys114 and Arg290 (2.94 Å), Tyr9 and the Gln286/Asn259 pair (2.85, 2.88 Å), and Arg12 and Asp288 (2.87, 3.09 Å) (Figure 9H).

**FIGURE 9:**
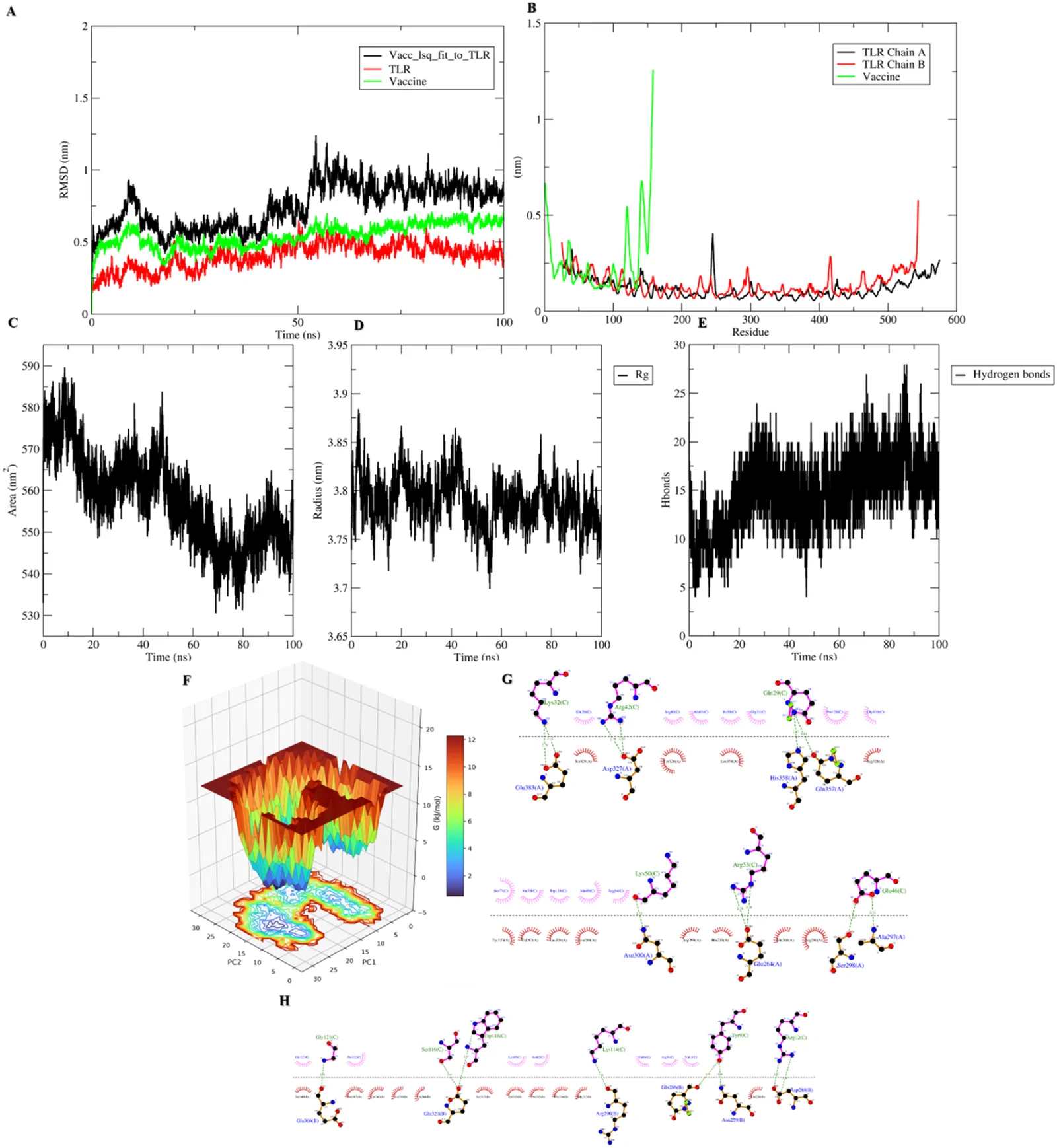
Molecular dynamics analysis of the TLR1/2 vaccine construct complex over a 100-ns simulation. **(A)** RMSD profiles of the TLR1/2 backbone, vaccine construct backbone, and vaccine backbone after alignment to the TLR1/2 backbone. **(B)** RMSF profiles of TLR1/2 chain A, TLR1/2 chain B, and the vaccine construct chain. **(C)** Solvent-accessible surface area (SASA) of the complex. **(D)** Radius of gyration (Rg) of the complete complex. **(E)** Number of hydrogen bonds formed between the TLR1/2 and vaccine construct during the simulation. **(F)** Free energy landscape projected onto the first two principal components, PC1 and PC2. **(G)** LigPlot + Interaction diagram of the vaccine construct with TLR1/2 chain A. **(H)** LigPlot + interaction diagram of the vaccine construct with TLR1/2 chain B.

### 3.10 Immune stimulation

The C-IMMSIM online platform was used in this study for the stimulation of immunity by administering a single dose of the immunogenic *in silico*, and this was subsequently measured. Following the single-dose antigen injection, antigen concentration peaked sharply at approximately 700,000 units/mL around day 1 and was cleared to near baseline levels by approximately day 5, consistent with rapid antigen processing and clearance. The combined IgM+IgG titers rose from day 5, reaching a peak of approximately 620,000 units (antigen count scale) corresponding to roughly 12,000+ on the antibody titer axis around day 13–15, before declining gradually but within the 33-day simulation window. The IgM component alone followed a similar but lower trajectory, peaking near day 14 at approximately 500,000 units and declining thereafter. The IFN-γ concentration rose steeply from day 0, reached its highest sustained level around day 8–14, and declined gradually thereafter without returning fully to baseline within the 33-day window, indicating a prolonged, rather than purely transient, interferon response. B-cell population dynamics showed an early transient decrease in the total B-cell count over the first ∼5 days, coinciding with the initial antigen exposure, followed by sustained expansion to a plateau of approximately 550 cells/mm^3^–600 cells/mm^3^ by day 10–15 that was maintained through the end of the simulation. A distinct memory B-cell subpopulation accumulated progressively over the same window and did not decline by day 33, consistent with durable humoral memory formation. Helper T cell (TH) total population rose steadily from baseline to a plateau of approximately 5,500 cells/mm^3^–6,000 cells/mm^3^ by day 15–20 and remained near that level through day 33. TH memory cells accumulated in parallel and persisted at a substantial fraction of the total population through the end of the simulation window, indicating sustained CD4^+^ memory formation. Subtype breakdown (inset plots) indicates a predominantly Th1-skewed response, consistent with the sustained IFN-γ signal. Cytotoxic T cell (TC) total population, by contrast, fluctuated within a comparatively narrow range (approximately 1,080 cells/mm^3^–1,155 cells/mm^3^) across the full simulation window, without a clear sustained expansion phase. The TC memory trace remained close to zero throughout, showing no distinguishable accumulation of memory subpopulation over the 33-day window. This is a materially different pattern from the TH compartment in the same simulation (Figure 10).

**FIGURE 10:**
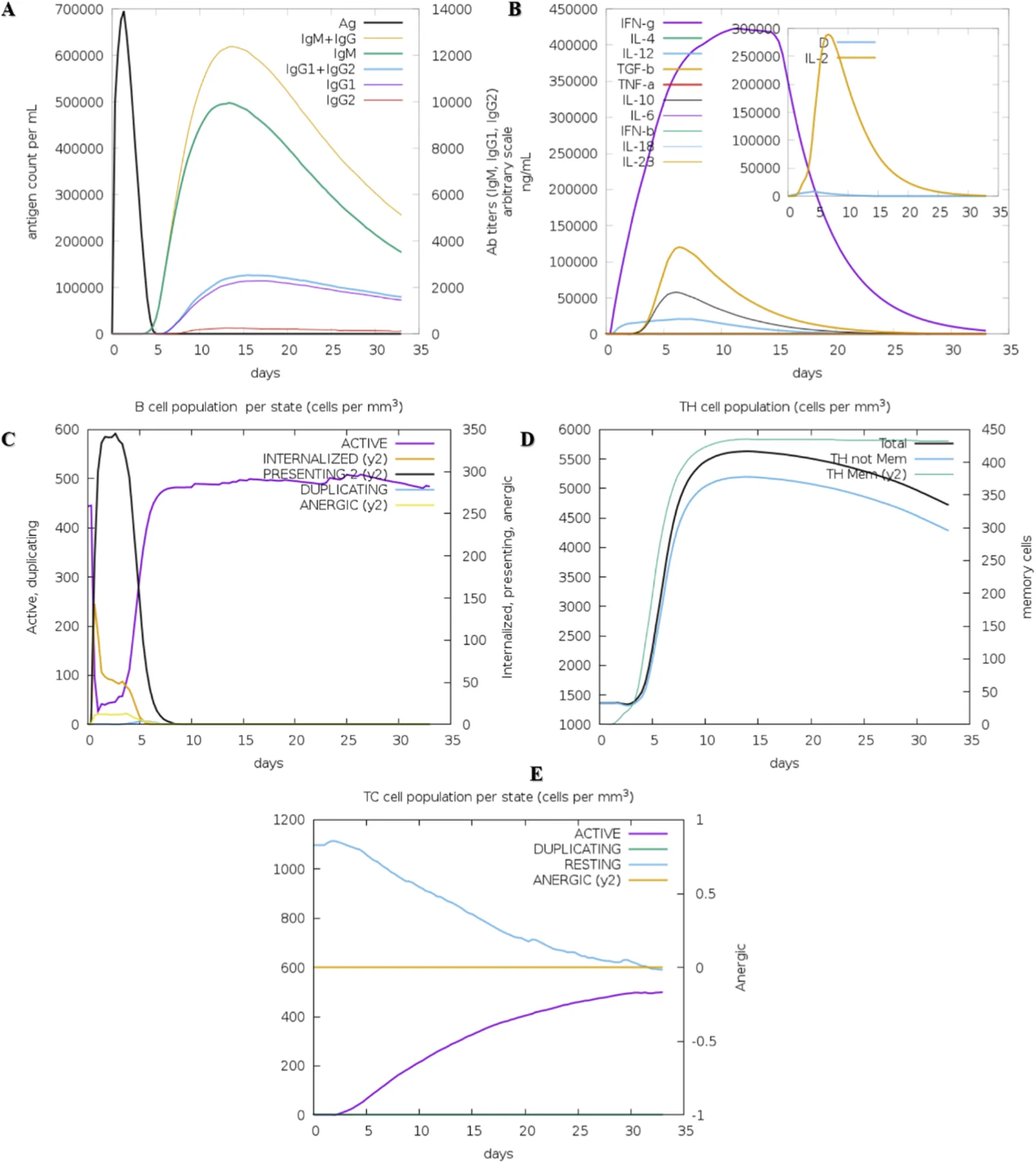
Immunization simulated with C-ImmSim. **(A)** Virus, immunoglobulins, and immunocomplexes. IgM and IgG levels clearly increased, while IgG2 levels increased less obviously. **(B)** IFN-g levels increased notably after stimulation. **(C)** B memory (y2) and B-isotype IgM levels increased. **(D,E)** TH memory (y2), TH, and TC cells generally increased.

### 3.11 Codon optimization and *in silico* vaccine construct design

The CAI values following the JCAT optimization improved from 0.85 to 0.96, and the GC content of the coding region changed from 71.06% to 69.79% (as defined in Methods); non-poly (A) and full transcript GC were 62.19% and 54.98%, respectively, for the optimized sequence. The 11 synonymous substitutions applied (Methods) substantially improved the start codon accessibility. The marginal unpaired probability at the AUG start codon rose from 0.00338 to 0.767 (−50/+100 nt context), and the local start region MFE improved from −34.4 to −22.6 kcal/mol. The observed/expected ratios of CpG (0.991–0.988) and UpA (0.824–0.810) remained near expected in both sequences. In the optimized construct, the five exact repeat families were identified and corresponded to the repeated GPGPG/AAY linker motifs rather than incidental duplication. Two donor sites below the 0.50 reference threshold, one acceptor site below 0.20, and additional acceptor site at position 128 registered at the 0.20 threshold borderline signal rather than a clear risk. These were identified by the cryptic splice-site analysis. This *in silico* assembly produced a coherent pIVTRup-based design containing one T7 promoter and one complete optimized transcript cassette (Figure 11A), consistent with a PCR-based strategy for generating a linear IVT template. The replacement of the native pIVTRup cassette with the optimized 1,035 bp mRNA cassette (Methods) generated a 4,944-bp recombinant plasmid, retaining a single T7 promoter and no duplicated vector-derived UTR or poly(A) sequence (Figure 11B). *In silico* verification confirmed the optimized cassette as one continuous transcription unit downstream of the promoter. This assembly supports the PCR-based linear IVT template strategy described in Methods. These findings support the feasibility of the proposed architecture but do not demonstrate successful amplification, transcription, capping, RNA integrity, or biological expression; no experimental cloning, PCR, *in vitro* transcription, or capping has been performed at this stage.

**FIGURE 11:**
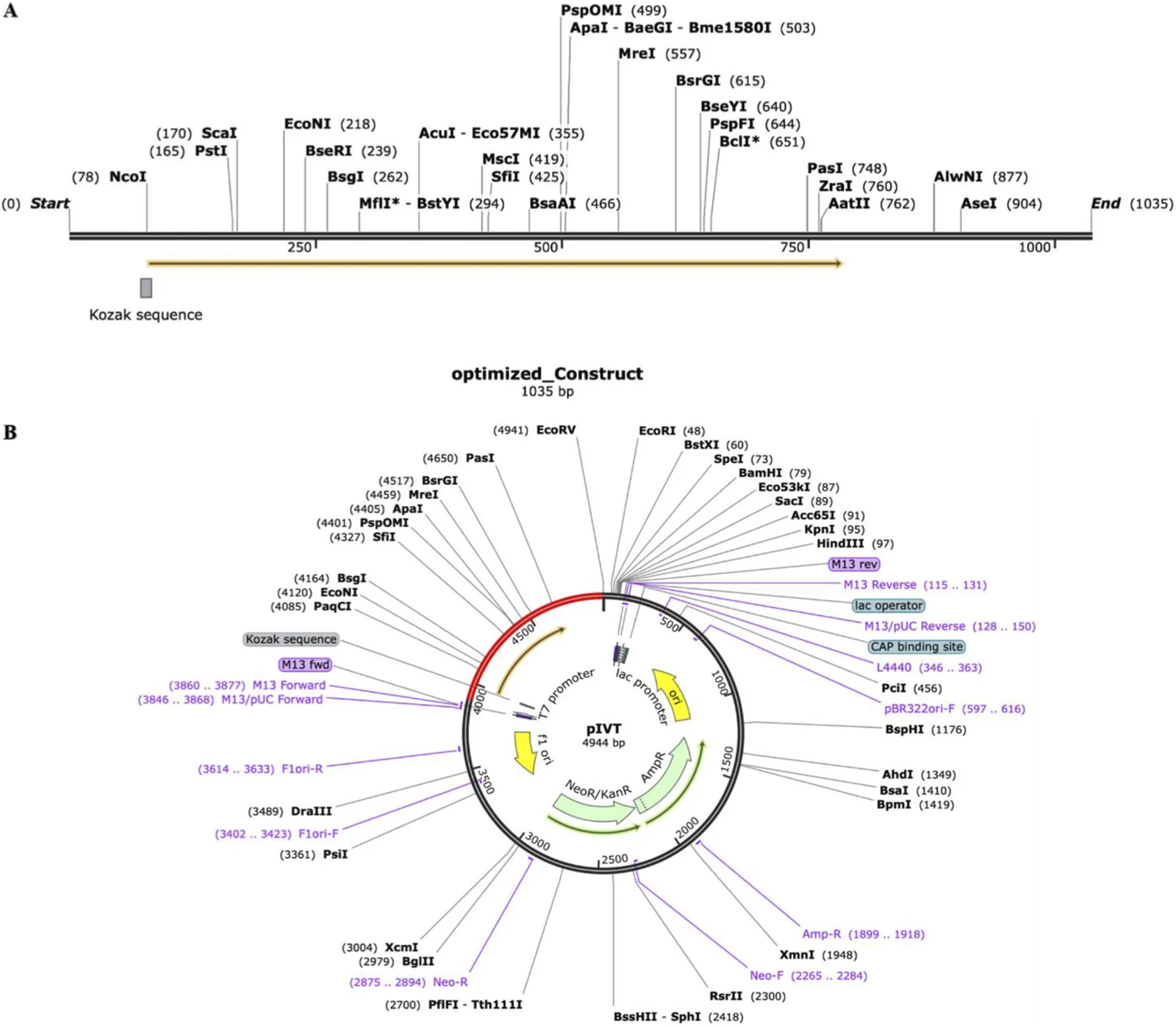
**(A)** Linear map of the optimized 1,035-bp mRNA cassette. The Kozak/start region is located near nucleotide 78, and the coding feature extends through approximately nucleotide 784. Restriction sites are shown for sequence verification. **(B)** Circular map of the 4,944-bp recombinant pIVTRup-derived plasmid. The optimized transcript cassette is positioned downstream of a single T7 promoter. Restriction sites are displayed for *in silico* construct verification and were not used as the primary IVT-template preparation strategy.

## 4 Discussion

HEV does not have a globally authorized vaccine despite its substantial annual burden, and HEV 239 lacks WHO prequalification. mRNA platforms offer scalability and modular design that were well-demonstrated during COVID-19 (Baden et al., 2021; Pardi et al., 2020). In this study, we apply the approach to a cross-genotype HEV ORF2 consensus construct, addressing a gap that current licensed and experimental HEV vaccines have not closed. ORF2 was selected for its established immunodominance and status as the primary target of neutralizing antibodies (Samavia et al., 2022), consistent with prior *in silico* HEV vaccine work (Lu et al., 2024; Ikram et al., 2023). The finalized epitope set showed strong conservancy overall, with residual variation concentrated specifically in genotype 1 rather than spread evenly, which is a refinement of the conservancy claim rather than a weakness in it. The tPA signal peptide and MITD domain serve distinct, non-redundant roles: tPA directs translocation through the secretory pathway (Ahmed et al., 2025), while MITD targets the construct to the MHC I compartment for CTL cross-presentation. Because both are cleavable signal elements rather than components of the mature antigen, structural characterization (secondary and tertiary modeling, refinement, and validation) was restricted to the construct core, while physicochemical properties (ProtParam) were assessed for the complete translated construct, consistent with comparable mRNA vaccine design precedent (Sira et al., 2025). MITD was retained at the C-terminus, downstream of the complete epitope core, consistent with its original characterization: fusing an antigen to this trafficking signal at the C-terminus, alongside an N-terminal signal peptide, was shown to substantially improve MHC class-I and class-II epitope presentation by mimicking the native recycling behavior of MHC class-I molecules between endosomal compartments (Kreiter et al., 2008). This same C-terminal architecture has since been directly validated in an mRNA vaccine platform, where MITD-containing constructs elicited significantly higher neutralizing antibody titers, expanded antigen-specific memory B cells, and stronger CD4+/CD8+ T-cell cytokine responses than otherwise identical constructs lacking MITD, with no adverse safety finding (Zhang et al., 2025). This ensures that the complete translated fusion protein, rather than a truncated intermediate, is routed through this pathway. Structural confidence in the AlphaFold3 model was lower than a that in comparable AlphaFold2-modeled HEV construct, consistent with the known difficulty of modeling flexible, linker-rich multi-epitope proteins that lack a single folded domain, and inter-domain positioning in this model should accordingly be interpreted with caution. Local stereochemical quality after refinement was the highest of the four constructs compared (97.44% Ramachandran-favored, versus 89.3%–93.2% for the three prior designs) (Lu et al., 2024; Ikram et al., 2023; Kumar et al., 2024). The docked complex showed a substantial interface (935.0 Å^2^, 51 total interface residues) stabilized by nine hydrogen bonds and three unique salt bridge pairs. The PISA-predicted interface solvation energy change was not statistically distinguished from background contacts (*P* = 0.476), consistent with the interpretation applied to interface energetics throughout this study: a real, sizeable interface, but not evidence of a specific biological interaction by itself. Normal mode analysis of the complex is presented as a coarse structural descriptor of the docked pose. The 100-ns MD simulation addresses the central limitation of the NMA analysis: a stable elastic network prediction does not, by itself, demonstrate that an interface persists under explicit solvation and thermal motion. Here, the interface’s hydrogen bond count increased and SASA decreased over the trajectory, and MM-GBSA returned a favorable net binding free-energy (−91.96 kcal/mol), together indicating that the docked pose is not an artifact of the initial minimization. At the same time, the vaccine chain’s continued RMSD drift after 70 ns and its RMSF peaks in the linker-adjacent regions corroborate, rather than contradict, the AlphaFold3-based disorder assessment: the construct core behaves as a flexible, multi-domain assembly that reorients around a comparatively rigid receptor surface rather than adopting one fixed docked geometry. This is consistent with the cautious, exploratory framing already applied to the TLR1/TLR2 docking throughout this study. Population coverage, at a global level, was achieved from a markedly smaller epitope set than comparable prior designs (seven epitopes vs. 23 in Lu et al. and Kumar et al.) and fell below both comparators’ reported global coverage (90.60% and 86.85%, respectively). Population coverage optimization formally involves a joint tradeoff between epitope panel size, total coverage, and regional coverage variance (Bui et al., 2006). The present construct minimizes the epitope panel size at a cost to the latter two, rather than achieving all three simultaneously, and this is reported here as a direct design tradeoff rather than reframed as an efficiency advantage. Regional variation, most severe in South Africa (22.16%) and Southwest Asia (60.84%), traces directly to the finalized MHC I panel’s exclusive HLA-B restriction, which also plausibly explains the immune simulation’s more limited cytotoxic T-cell expansion relative to the helper T cell and humoral response; both findings point to the same design constraint and warrant confirmation via CD8^+^ functional assays rather than simulation alone. Extending MHC I coverage to HLA-A and HLA-C alleles is identified as the direct lever for addressing this constraint in a subsequent design iteration. A total of 36 human peptides across 27 genes retained predicted binding to the same HLA allele as their corresponding viral epitope; nine bound at least as strongly as the viral epitope itself. Shared residues among these nine ranged from 7 out of 9 (ABCB8, the sole MHC class-I match) to 6–8 out of 15 (the eight MHC class-II matches, each requiring flanking-sequence reconstruction) (Supplementary Data), none exceeding the nine-residue MHC class II binding core. This bears comparison to a documented clinical case of fatal T-cell cross-reactivity in a cancer immunotherapy trial, where two proteins sharing just five of nine residues (56% identity) were structurally indistinguishable to engineered T-cells once bound to MHC (Mendes et al., 2022); the identity seen here cannot, on that precedent, be interpreted as reassuring. Retained or superior binding is not confirmed cross-reactivity, as TCR recognition depends on more than linear sequence and HLA-binding residues. Two matches warrant individual note: an exact 7-mer match to human apelin, a non-binder for two of three HLA-II alleles, and a one-mismatch ABCB8-derived match retaining HLA-B*40:01 binding. Triple stop codons were included to limit ribosomal read-through under pseudouridine modification. Codon optimization relieved start-codon secondary structure at the cost of a small GC reduction, but one cryptic splice-site signal remained at the reference confidence threshold rather than clearly below it. The proposed pIVTRup-based production strategy cassette replacement, PCR-amplified linear template, and downstream capping remains an *in silico* design; no experimental cloning, transcription, or capping has been performed. These findings are computational predictions throughout and should not be read as evidence of actual protective efficacy in a host organism; translating this construct from hypothesis to experimental candidate requires a defined validation sequence. Computationally, the remaining priorities are extending the MHC I panel to include HLA-A and HLA-C alleles to address the regional coverage gap identified above and reassessing structural confidence as AlphaFold3 refinement continues. Experimentally, the proposed sequence is: (1) PCR amplification and sequence verification of the linear IVT template from the recombinant pIVTRup plasmid; (2) *in vitro* transcription, capping, and RNA quality testing; (3) protein expression verification (Western blot or mass spectrometry); (4) HLA binding assays, including confirmation of the HLA-B restricted MHC I panel and testing whether additional epitopes could extend HLA-A/C coverage; (5) antigen processing assays confirming proteasomal processing and TAP-dependent presentation; (6) PBMC cytokine assays with CD8^+^ functional readouts (e.g., IFN-γ ELISpot) to test the limited cytotoxic T-cell expansion seen *in silico*; (7) antibody binding assays against native ORF2; (8) neutralization assays across HEV genotypes 1–4; (9) animal immunogenicity studies. Without this sequence, the present findings remain a computational hypothesis rather than a validated candidate.

## 5 Conclusion

In this study, we present a computationally designed multi-epitope mRNA vaccine candidate targeting hepatitis E virus genotypes 1–4, developed using an immunoinformatics-based workflow. By deriving epitopes from a cross-genotype ORF2 consensus sequence (660 amino acids), the construct is intended to address the narrow genotypic and geographic coverage of the sole licensed vaccine, HEV 239. Seven epitopes four MHC I, two MHC II, and one B-cell were selected through multi-parameter computational screening for antigenicity, non-allergenicity, non-toxicity, IFN-γ induction, pre-genotype conservancy ranging from 80% to 100%, and the absence of a priority-level human proteome cross-reactivity signal. The construct (235 amino acids total, comprising a tPA signal peptide, β-defensin adjuvant, EAAAK linker, epitope core, and MITD-trafficking domain) showed predicted antigenicity. Structural modeling of the construct core indicated favorable local stereochemical quality but limited global structural confidence, reflecting the substantial predicted disorder typical of flexible, linker-rich multi-epitope constructs. Docking against the TLR1/TLR2 heterodimer, selected on adjuvant-specific mechanistic grounds, identified an interface stabilized by multiple hydrogen bonds and salt bridges; normal mode analysis of the docked complex is presented as a coarse structural descriptor, and a subsequent 100-ns molecular dynamics simulation supported a persistent, increasingly hydrogen-bonded interface with a favorable MM-GBSA binding free-energy (−91.96 kcal/mol), while also corroborating construct core flexibility already indicated by the structural modeling. *In silico* immune simulation predicted a sustained humoral response and durable helper T cell memory, with more limited evidence of cytotoxic T cell expansion attributable, at least in part, to the finalized epitope set’s restriction to HLA-B alleles. The predicted global and South Asian population coverage of 75.87% and 62.82% is lower than two comparable prior HEV multi-epitope designs and reflects the tradeoff of a substantially smaller, HLA-B-restricted epitope panel; extending MHC I coverage to HLA-A and HLA-C alleles is identified as a priority for a subsequent design iteration rather than the present construct’s strength. These findings constitute a computational hypothesis. Experimental validation, including RNA production and quality testing, protein expression verification, HLA binding assays, antigen processing assays, PBMC-based cytokine assays with CD8^+^ functional readouts, antibody binding and neutralization assays, and animal immunogenicity studies, is required before immunogenicity, safety, or protective efficacy can be inferred.

## Data Availability

The original contributions presented in the study are included in the article/Supplementary Material; further inquiries can be directed to the corresponding authors.
