# Supplementary material for: Immunoinformatics-based design of a multi-epitope mRNA vaccine candidate targeting the major human-infecting hepatitis E virus genotypes 1–4
Source: Front Bioinform. 2026 Sep 23;6:1922523. doi: 10.3389/fbinf.2026.1922523 (PMC13642824; doi:10.3389/fbinf.2026.1922523)
Supplement: Supplementary file 1 [file Table1.docx]

Supplementary Data

| Feature | Ikram et al. (2023) | Lu et al. (2024) | Kumar et al. (2024) | Present study |
| --- | --- | --- | --- | --- |
| Target protein(s) | ORF2 only | ORF2 + ORF3 | ORF2+ORF3+ORF1(polyprotein) | ORF2 only |
| Genotype basis | Consensus, GT1/3/4 | Individual seqs, GT1/3/4 | Single reference accessions | Consensus, GT1-4 |
| Total epitopes | 41 | 23 | ~23 | 7 |
| Global population coverage | Not reported | 90.60% | 86.85% | 75.87% |
| Regional breakdown | Not reported | Not reported | Yes - 5 regions | Yes - 16 regions |
| Adjuvant | Beta-defensin (45 aa) | Beta-defensin 3 + LL37 | Beta-defensin (45 aa) | Beta-defensin (45 aa) |
| Construct length | Not stated | 433 aa | 453 aa | 235 aa |
| 3D structural tool | I-TASSER | AlphaFold2 (pLDDT 75.45) | I-TASSER | AlphaFold3 (pLDDT 54.3) |
| Ramachandran favored | 89.3% | 93.2% | 91.1% | 97.44% |
| Docking receptor(s) | TLR3 | TLR3 | TLR3+TLR4 | TLR1/TLR2 heterodimer |
| Stability method | Molecular dynamics (GROMACS) | Molecular dynamics (GROMACS, 30ns) | Molecular dynamics (NAMD, 100ns) | NMA (iMODS) + molecular dynamics (GROMACS, 100 ns) + MM-GBSA |
| Immune simulation | Not reported | C-ImmSim | C-ImmSim | C-IMMSIM |
| Experimental validation | None | None | None | None |

**Table S1: Comparator Analysis Against Prior HEV Multi-Epitope Vaccine Designs**

**Table S2: HEV ORF2 Dataset Curation: Inclusion/Exclusion Criteria and Composition.**

| Panel A: Inclusion/exclusion criteria | | |
| --- | --- | --- |
| Category | **Criterion** | |
| Inclusion | HEV ORF2 protein sequence assigned to genotype 1, 2, 3, or 4 | |
| Inclusion | Sequence derived from a human (Homo sapiens) isolate | |
| Inclusion | ORF2 sequence length between 600 and 680 amino acids | |
| Inclusion | Retrievable accession number with sufficiently defined annotation | |
| Inclusion | Manually reviewed UniProt reference sequences, eligible after duplicate checking | |
| Exclusion | Non-human host or inconsistent host information | |
| Exclusion | Out-of-range ORF2 length (<600 or >680 aa) | |
| Exclusion | Missing, ambiguous, or unsupported genotype assignment | |
| Exclusion | Duplicate accession or duplicate cross-database reference entry | |
| Exclusion | Uncharacterized or inadequately annotated record | |
| Panel B: Final composition by genotype | | |
| Genotype | **Sequences** | |
| 1 | 23 | |
| 2 | 3 | |
| 3 | 319 | |
| 4 | 95 | |
| Total | **440** | |
| Panel C : Geographic distribution (all 440 sequences) | | |
| Country/region | | **Count** |
| Burma | | 1 |
| Cambodia | | 1 |
| China | | 12 |
| France | | 131 |
| Germany | | 5 |
| Hungary | | 1 |
| India | | 1 |
| Italy | | 1 |
| Japan | | 174 |
| Kyrgyzstan | | 3 |
| Mexico | | 2 |
| Mongolia | | 2 |
| Myanmar | | 1 |
| Nepal | | 5 |
| New Caledonia | | 3 |
| Nigeria | | 1 |
| Pakistan | | 1 |
| Spain | | 73 |
| Sweden | | 6 |
| Thailand | | 5 |
| United Kingdom | | 5 |
| United States | | 1 |
| Unknown | | 2 |
| Uruguay | | 1 |
| USA | | 2 |

**Figure F1: MHC and B-cell Epitope Filtering Funnel Flowcharts**

**Table S3:MHC and B-cell Epitope Filtering Funnel.**

| MHC Epitope Filtering Funnel | | | |
| --- | --- | --- | --- |
| Stage | **Decision rule** | **Retained** | **Removed at stage** |
| Starting set | Unique exact SB peptide sequences | 481 | - |
| AlgPred2 | Model 2; threshold 0.30; Non-Allergen | 86 | 395 |
| ToxinPred3 | Model 2; threshold 0.38; Non-Toxin | 84 | 2 |
| PEPMatch | 0 mismatch k=5 exact / 1 mismatch k=3 near | 84 | 0 |
| VaxiJen | Virus model; threshold >=0.40 | 66 | 18 |
| B-Cell Epitope Filtering Funnel | | | |
| Stage | **Rule** | **Retained** | **Removed at stage** |
| Starting set | 99 unique linear candidates (ElliPro 13 / BepiPred2 22 / ABCpred 64) | 99 | - |
| AlgPred2 | Non-Allergen; Model 2; threshold 0.30 | 23 | 76 |
| ToxinPred3 | Non-Toxin; ET+MERCI Model 2; threshold 0.38 | 22 | 1 |
| VaxiJen | ANTIGEN; Virus mode; threshold 0.40 (too-small counted as fail) | 16 | 6 |

**Table S4:ORF2 Structural Domain Mapping, Final Epitope Set**

| Epitope | Type | Position | ORF2 domain (PDB 2ZTN) |
| --- | --- | --- | --- |
| ILRRQYNL | MHC-I | 131-138 | Shell (S), 129-319 |
| IMATEASNY | MHC-I | 174-182 | Shell (S), 129-319 |
| GEVGRGIALTL | MHC-I | 362-372 | Middle (M), 320-455 |
| FSVLRANDVLW | MHC-I | 462-472 | Protruding (P), 456-606 |
| KTFYVLPLRGKLSFW | MHC-II | 534-548 | Protruding (P), 456-606 |
| SVLRANDVLWLSLTA | MHC-II | 463-477 | Protruding (P), 456-606 |
| EYDQTTYGSSTNPMY | B-cell | 479-493 | Protruding (P), 456-606; within neutralizing region |

**Table S5: B-Cell Candidates Passing All Mandatory Filters: Selected vs. Deprioritized**

| Criterion | EYDQTTYGSSTNPMY (selected) | QTTTTPTSVDMNSITS (deprioritized) |
| --- | --- | --- |
| Position (ORF2) | 479-493 | 214-229 |
| ORF2 structural domain | Protruding (P), 456-606 | Shell (S), 129-319 |
| Neutralization-region overlap | Within region | 0% - no overlap |
| ElliPro (>=0.50 pass) | 0.799, 100% residues - PASS | 0.415, 25% residues - FAIL |
| BepiPred-2.0 (>=0.50 pass) | 0.574, 100% - PASS | 0.581, 100% - PASS |
| ABCpred (>=0.51 pass) | 0.94, 100% - PASS | 0.88, 100% - PASS |
| Karplus-Schulz flexibility (>=1.00 pass) | 1.043, 80% - PASS | 1.042, 75% - PASS |
| DiscoTope-3.0 (>=50% epitope-residue pass) | -0.135, 20% - FAIL | 0.007, 12.5% - FAIL |
| NetSurfP RSA (>=50% pass) | 0.387, 86.7% - PASS | 0.427, 75% - PASS |
| CABS-FLEX (>=1.00 A pass) | 1.111, 53.3% - PASS | 0.856, 18.8% - FAIL |
| Combined evidence score | 6/7 criteria; 9/10 filters | 4/7 criteria; 7/10 with filters |
| VaxiJen / AlgPred2 / ToxinPred3 | ANTIGEN / Non-Allergen / Non-Toxin - all PASS | ANTIGEN / Non-Allergen / Non-Toxin - all PASS |
| Exact-reference conservancy | 80.0% | 93.8% |

**Table S6: Predicted Linear B-Cell Epitope Selected From The Consensus ORF2 Sequence Of Hepatitis E Virus (HEV) Genotypes 1–4**

| Epitope Type | Peptide Sequence | Length | Position (ORF2) | Supporting Prediction Criteria 7 |
| --- | --- | --- | --- | --- |
| Linear B cell located within the reported neutralizing region of ORF2 and showed 80% sequence conservancy among the analyzed HEV genotypes (1–4) | EYDQTTYGSSTNPMY | 15 | 479-493 | ElliPro, BepiPred-2.0, ABCPred, Karplus–Schulz Flexibility, NetSurfP 3.0 and CABS-FLEX and DiscoTope-3 |

**Table S7: Human Peptides Retaining Binding To The Same HLA Allele As The Corresponding Viral Epitope, At Comparable Or Greater Predicted Strength**

| Parent epitope | Class | HLA allele | Viral segment | Human peptide | Substitution | Length | Gene | Viral binding | Human binding |
| --- | --- | --- | --- | --- | --- | --- | --- | --- | --- |
| GEVGRGIALTL | I | HLA-B*40:01 | EVGRGIA | ELGRGIA | V2L | 7 | ABCB8 | %rank 0.08 (SB) | %rank 0.08 (SB) |
| SVLRANDVLWLSLTA | II | HLA-DRB1*13:02 | SVLRAND | SVLRANM | D7M | 7 | PARD3 | 9.1 nM (SB) | 1.9 nM (SB) |
| KTFYVLPLRGKLSFW | II | HLA-DRB1*01:01 | LPLRGKL | LSLRGKL | P2S | 7 | DZIP1L | 4.8 nM (SB) | 2.3 nM (SB) |
| KTFYVLPLRGKLSFW | II | HLA-DRB1*01:01 | VLPLRGK | VLRLRGK | P3R | 7 | LACTBL1 | 4.8 nM (SB) | 3.0 nM (SB) |
| SVLRANDVLWLSLTA | II | HLA-DRB1*13:02 | LRANDVL | LRANRVL | D5R | 7 | UPK3BL1 | 9.1 nM (SB) | 3.5 nM (SB) |
| SVLRANDVLWLSLTA | II | HLA-DRB1*13:02 | LRANDVL | LRANRVL | D5R | 7 | UPK3BL2 | 9.1 nM (SB) | 3.5 nM (SB) |
| KTFYVLPLRGKLSFW | II | HLA-DRB1*01:01 | PLRGKLS | PLRGLLS | K5L | 7 | PIGV | 4.8 nM (SB) | 4.1 nM (SB) |
| SVLRANDVLWLSLTA | II | HLA-DRB1*13:02 | LRANDVL | LRANQVL | D5Q | 7 | BRAP | 9.1 nM (SB) | 7.3 nM (SB) |
| KTFYVLPLRGKLSFW | II | HLA-DQA1*05:01/DQB1*04:02 | VLPLRGK | VHPLRGK | L2H | 7 | LRP1 | 26.9 nM (SB) | 15.9 nM (SB) |

**Table S8: Sub Peptide Human Proteome Homology Assessment**

| Epitope | Type | 9-mer exact match | 8-mer exact match | 7-mer exact match | One mismatch match | Priority mimicry signal |
| --- | --- | --- | --- | --- | --- | --- |
| ILRRQYNL | MHC-I | None | None | None | Not flagged | No |
| IMATEASNY | MHC-I | None | None | None | Yes, limited overlap | No |
| GEVGRGIALTL | MHC-I | None | None | None | Yes (ABCB8-derived, HLA-B*40:01) | No, secondary HLA-binding reassessment confirmed the human peptide retains predicted binding to HLA-B*40:01 but reported as a limitation requiring experimental confirmation |
| FSVLRANDVLW | MHC-I | None | None | None | Not flagged | No |
| KTFYVLPLRGKLSFW | MHC-II | None | None | None | Yes, limited overlap | No |
| SVLRANDVLWLSLTA | MHC-II | None | None | LWLSLTA (human apelin) | Yes | No, secondary HLA-binding reassessment showed the human peptide as a non-binder for two of three HLA-II alleles.and a weak binder for the third (predicted IC50 13.8 nM versus 5.1 nM for the viral peptide) |
| EYDQTTYGSSTNPMY | B-cell | None | None | None | Not flagged | No |

**Table S9: Per Genotype Conservancy of Finalized Epitopes**

| Epitope | Type | Genotype 1 (N=5, Q81871, Q04611, Q68985, P29326, P33426) | Genotype 2 (N=1, Q03500) | Genotype 3 (N=1, Q9YLQ9) | Genotype 4 (N=1, Q9IVZ8) |
| --- | --- | --- | --- | --- | --- |
| ILRRQYNL | MHC-I | 100% | 100% | 100% | 100% |
| IMATEASNY | MHC-I | 100% | 100% | 100% | 100% |
| GEVGRGIALTL | MHC-I | 90.91% (10/11), V3I in 5/5 refs | 100% | 100% | 100% |
| FSVLRANDVLW | MHC-I | 100% | 100% | 100% | 100% |
| KTFYVLPLRGKLSFW | MHC-II | 93.33% (14/15) in 4/5 refs: Y4F; 86.67% (13/15) in 1/5 refs: T2I+Y4F | 93.33% (14/15): Y4F | 100% | 100% |
| SVLRANDVLWLSLTA | MHC-II | 100% | 100% | 100% | 100% |
| EYDQTTYGSSTNPMY | B-cell | 80.00% (12/15) in 4/5 refs: T5S/N12G/M14V; in 1/5: T5S/N12A/M14V | 80.00% (12/15): T5S/N12G/M14V | 100% | 100% |

**Table S10: AIPpred Anti-Inflammatory Peptide Classification**

| Epitope | Type | AIP / Non-AIP | Probability |
| --- | --- | --- | --- |
| ILRRQYNL | MHC-I | AIP | 0.4372 |
| IMATEASNY | MHC-I | AIP | 0.4349 |
| GEVGRGIALTL | MHC-I | AIP | 0.4209 |
| FSVLRANDVLW | MHC-I | AIP | 0.4372 |
| KTFYVLPLRGKLSFW | MHC-II | AIP | 0.3849 |
| SVLRANDVLWLSLTA | MHC-II | AIP | 0.3860 |
| EYDQTTYGSSTNPMY | B-cell | AIP | 0.4442 |

**Table S11: Predicted global and regional population coverage of the selected MHC class I and II epitopes.**

| Population/Area | Coverage (%) | Average Hit | PC90 |
| --- | --- | --- | --- |
| Central Africa | 65.07 | 0.92 | 0.29 |
| Central America | 88.3 | 1.46 | 0.85 |
| East Africa | 75.71 | 1.20 | 0.41 |
| East Asia | 71.66 | 1.12 | 0.35 |
| Europe | 77.31 | 1.26 | 0.44 |
| North Africa | 65.93 | 0.94 | 0.29 |
| North America | 87.39 | 1.56 | 0.79 |
| Northeast Asia | 71.24 | 1.07 | 0.35 |
| Oceania | 80.59 | 1.34 | 0.52 |
| South Africa | 22.16 | 0.24 | 0.13 |
| South America | 77.74 | 1.23 | 0.45 |
| South Asia | 62.82 | 0.88 | 0.27 |
| Southeast Asia | 72.49 | 1.09 | 0.36 |
| Southwest Asia | 60.84 | 0.80 | 0.26 |
| West Africa | 82.58 | 1.30 | 0.57 |
| West Indies | 73.88 | 1.16 | 0.38 |
| World | 75.87 | 1.22 | 0.41 |
| Average | **71.27** | **1.11** | **0.42** |
| Standard Deviation | **14.45** | **0.29** | **0.18** |

**Table S12: The physiochemical properties of the vaccine construct.**

| **Molecular weight** | | 25004.02 |
| --- | --- | --- |
| **Theoretical pI** | | 9.61 |
| **Total number of atoms** | | 3523 |
| **Formula** | | C1102H1773N315O318S15 |
| **Instability index** | | 39.40 |
| **Aliphatic index** | | 88.47 |
| **Grand average of hydropathicity [GRAVY]** | | 0.087 |
| **Estimated half- life:** | **Mammalian Reticulocytes [In vitro]** | 30 hours |
|  | **Yeast [In vivo]** | >20 hours |
|  | **Escherichia coli [In vivo]** | >10 hours |

**mRNA vaccine construct**

5′ m7GCap– 5′ UTR–Kozak sequence–Signal peptide (tPA) -Adjuvant-peptide vaccine sequence-MITD sequence- Stop codons- 3’ UTR-Poly (A) tail.

**5'm7G Cap**

**5'UTR**

NCA-7d (Derived from NCA-7)

GGCAAAAAUCAAAAUCAAUCAUCAUCACAACAUCAACAAUCAAUCAUCAACACAUCAUCAAGACACCACCGCCGCCGCCAUGGCG

**Kozak Sequence**

GCCGCC(A/G)(C/A)CAUGGCG

GCCGCCGCCAUGGCG

**Signal Peptide**

>sp|P00750|TPA_HUMAN Tissue-type plasminogen activator

AUGGACGCCAUGAAGAGGGGCCUGUGCUGCGUGCUGCUGCUGUGCGGCGCCGUGUUCGUGAGCCCC

**mRNA Sequence**

GGCAUCAUCAACACCCUGCAGAAGUACUACUGCAGGGUGAGGGGCGGCAGGUGCGCCGUGCUGAGCUGCCUGCCCAAGGAGGAGCAGAUCGGCAAGUGCAGCACCAGGGGCAGGAAGUGCUGCAGGAGGAAGAAGGAGGCCGCCGCCAAGAUCCUGAGGAGGCAGUACAACCUGGCCGCCUACGGCGAGGUGGGCAGGGGCAUCGCCCUGACCCUGGCCGCCUACUUCAGCGUGCUGAGGGCCAACGACGUGCUGUGGGCCGCCUACAUCAUGGCCACCGAGGCCAGCAACUACGGCCCCGGCCCCGGCAAGACCUUCUACGUGCUGCCCCUGAGGGGCAAGCUGAGCUUCUGGGGCCCCGGCCCCGGCAGCGUGCUGAGGGCCAACGACGUGCUGUGGCUGAGCCUGACCGCCGGCGGCGGCGGCAGCGAGUACGACCAGACCACCUACGGCAGCAGCACCAACCCCAUGUAC

**Complete Optimized mRNA:**

GGCAAAAAUCAAAAUCAAUCAUCAUCACAACAUCAACAAUCAAUCAUCAACACAUCAUCAAGACACCACCGCCGCCGCCAUGGACGCGAUGAAACGCGGGCUGUGUUGUGUGUUACUGCUUUGUGGAGCCGUGUUUGUGAGCCCCGGCAUCAUCAACACCCUGCAGAAGUACUACUGCCGCGUGCGCGGCGGCCGCUGCGCCGUGCUGAGCUGCCUGCCCAAGGAGGAGCAGAUCGGCAAGUGCAGCACCCGCGGCCGCAAGUGCUGCCGCCGCAAGAAGGAGGCCGCCGCCAAGAUCCUGCGCCGCCAGUACAACCUGGCCGCCUACGGCGAGGUGGGCCGCGGCAUCGCCCUGACCCUGGCCGCCUACUUCAGCGUGCUGCGCGCCAACGACGUGCUGUGGGCCGCCUACAUCAUGGCCACCGAGGCCAGCAACUACGGCCCCGGCCCCGGCAAGACCUUCUACGUGCUGCCCCUGCGCGGCAAGCUGAGCUUCUGGGGCCCCGGCCCCGGCAGCGUGCUGCGCGCCAACGACGUGCUGUGGCUGAGCCUGACCGCCGGCGGCGGCGGCAGCGAGUACGACCAGACCACCUACGGCAGCAGCACCAACCCCAUGUACAUCAUCGCCGGCCUGGUGCUGCUGGGCGCCGUGAUCACCGGCGCCGUGGUGGCCGCCGUGAUGUGGCGCCGCAACAGCAGCGACCGCAAGGGCGGCAGCUACAGCCAGGCCGCCAGCAGCGACAGCGCCCAGGGCAGCGACGUCAGCCUGACCGCCUGCAAGGUGUAAUGAUAGUUGUGUAUGCGUUAAUAAAAAGAAGGAACUCGUAAAAACUCAAUGUAUUUCUGAGGAAGCGUGGUGCAUAAUGCCACGCAGCGUCUGCAUAACUUUUAUUAUUUCUUUUAUUAAUCAACAAAAAAAAAAAAAAAAAAAAAAAAAAAAAAAAAAAAAAAAAAAAAAAAAAAAAAAAAAAAAAAAAAAAAAAAAAAAAAAAAAAAAAAAAAAAAAAAAAAAAAAAAAAAAAAAAAAAAAAAAAA

**MITD:**

AUCAUUGCCGGCCUGGUUCUGCUCGGAGCCGUGAUUACAGGUGCUGUGGUGGCUGCUGUGAUGUGGCGGAGAAACAGCAGCGACAGAAAAGGCGGCAGCUACUCUCAGGCCGCCAGCUCUGAUUCUGCCCAGGGCUCUGAUGUGUCUCUGACCGCCUGCAAGGUG

**3' UTR**

**S27a + R3U**

UUGUGUAUGCGUUAAUAAAAAGAAGGAACUCGUAAAAACUCAAUGUAUUUCUGAGGAAGCGUGGUGCAUAAUGCCACGCAGCGUCUGCAUAACUUUUAUUAUUUCUUUUAUUAAUCAACAAA

**Poly-A tail**

AAAAAAAAAAAAAAAAAAAAAAAAAAAAAAAAAAAAAAAAAAAAAAAAAAAAAAAAAAAAAAAAAAAAAAAAAAAAAAAAAAAAAAAAAAAAAAAAAAAAAAAAAAAAAAAAAAAAAAA

Table S13: RNA Regional Coordinates

| Region | Coordinates | Length |
| --- | --- | --- |
| 5′ region | 1-79 | 79 nt |
| ORF/CDS | 80-784 | 705 nt |
| 3′ region | 785-915 | 131 nt |
| Non-poly(A) | 1-915 | 915 nt |
| Terminal poly(A) | 916-1035 | 120 nt intended |
| Full transcript | 1-1035 | 1,035 nt |

**Calculations for mRNA analysis:**

**GC percentage**

GC (%) = 100 × [n(G)+n(C)]/L. ORF GC uses positions 80-784, non-poly(A) GC uses positions 1-915, and full-transcript GC uses positions 1-1035. The full-transcript value is descriptive because the A120 tract lowers it by design.

**Sliding-window GC**

GCwindow(s,W) = 100 × [n(G)+n(C) in s...s+W-1]/W. Windows of 30, 60 and 100 nt are advanced in 10-nt steps. An end-anchored final window is added when required. The primary count is the number of 100-nt windows with GC ≥70%.

**CpG and UpA**

f(XY)=N(XY)/(L-1), f(X)=N(X)/L, and O/E(XY)=f(XY)/[f(X)f(Y)]. Counts use overlapping adjacent pairs and are calculated independently within each region.

**RNA folding**

ViennaRNA Python 2.7.2 was used at 37 °C with dangles=2 and noLP=0. Normalized MFE=MFE/L. Marginal unpaired probability is Pu(i)=1-Σj≠i p(i,j).

**AUG accessibility**

AAUG=[Pu(A)+Pu(U)+Pu(G)]/3. The primary candidate-ranking metric uses a -50/+100 context. The -30/+60 value is a separate local-context result. Joint segment probabilities are separate from the marginal mean.

**Opening energy**

ΔGopen=-RT ln(Pjoint), with R=0.0019872041 kcal mol-1 K-1 and T=310.15 K.

**Competing AUGs**

Rintended:competitor=Aintended/Acompetitor. Ratios must use accessibility values computed in the same folding context.

**Human codon use**

python-codon-tables 0.1.18, h_sapiens_9606. Relative adaptiveness wi=fhuman(codoni)/max(fhuman(synonymous codons)).

**Splice-site prediction**

NetGene2 2.4 was run with the Homo sapiens model on the 915-nt non-poly(A) sequence.

Table S14: Baseline versus Optimized summary

| Metric | Baseline | Optimized |
| --- | --- | --- |
| Input length | 1,035 nt | 1,035 nt |
| Non-poly(A) analysis length | 915 nt | 915 nt |
| Terminal poly(A) | 120 nt | 120 nt |
| ORF length | 705 nt | 705 nt |
| ORF GC | 71.06% | 69.79% |
| Non-poly(A) GC | 63.17% | 62.19% |
| Full transcript GC | 55.85% | 54.98% |
| 100-nt GC range | 35-78% | 35-78% |
| 100-nt windows ≥70% | 43 | 42 |
| CpG count / O:E | 90 / 0.991 | 87 / 0.988 |
| UpA count / O:E | 25 / 0.824 | 26 / 0.810 |
| Exact repeat families | 5 | 5 |
| Summary tandem-repeat families | 0 | 0 |
| Homopolymers | 0 | 0 |
| Polyadenylation-like motifs | 6 | 6 |
| AUG accessibility, -30/+60 | 0.01805 | 0.69929 |
| AUG accessibility, -50/+100 | 0.00338 | 0.76700 |
| Start MFE, -30/+60 | -34.4 kcal/mol | -22.6 kcal/mol |

Table S15: Optimized RNA-structure outputs

| Metric | Optimized result |
| --- | --- |
| Complete non-poly(A) MFE | -389.9 kcal/mol |
| Normalized MFE | -0.4261 kcal mol-1 nt-1 |
| Ensemble free energy | -403.81 kcal/mol |
| Mean unpaired probability | 0.3763 |
| Full-transcript first-AUG marginal mean | 0.61345 |
| First 200 nt MFE | -50.1 kcal/mol |
| First 200 nt first-AUG marginal mean | 0.5837 |
| Start region -30/+60 MFE | -22.6 kcal/mol |
| AUG marginal mean in -30/+60 | 0.6993 |
| Candidate-table AUG marginal mean in -50/+100 | 0.7670 |
| A, U and G marginal unpaired probabilities | 0.7619, 0.6774, 0.6586 |
| -10/+15 mean accessibility | 0.4989 |
| -30/+30 mean accessibility | 0.4161 |
| Strongest early competitor | nt 89; reported accessibility 0.65662 |
| Other AUG competitors | 8 |

Table 16: Synonymous substitutions in Optimized

| Codon | AA | Original | New |
| --- | --- | --- | --- |
| 3 | A | GCC | GCG |
| 5 | K | AAG | AAA |
| 7 | G | GGC | GGG |
| 9 | C | UGC | UGU |
| 10 | C | UGC | UGU |
| 12 | L | CUG | UUA |
| 14 | L | CUG | CUU |
| 15 | C | UGC | UGU |
| 16 | G | GGC | GGA |
| 19 | F | UUC | UUU |
| 228 | V | GUG | GUC |

Table 17:NetGene2 predictions after the codon-228 substitution

| Site type | Position | Strand | Confidence | Threshold interpretation |
| --- | --- | --- | --- | --- |
| Donor | 761 | + | 0.31 | Below 0.50 donor reference threshold |
| Donor | 782 | + | 0.31 | Below 0.50 donor reference threshold |
| Acceptor | 128 | + | 0.2 | At 0.20 acceptor reference threshold |
| Acceptor | 141 | + | 0.17 | Below 0.20 acceptor reference threshold |

Table 18: Exact repeated segments in Optimized

| Length (nt) | Occurrences | Positions | Regions | Sequence |
| --- | --- | --- | --- | --- |
| 31 | 2 | 373, 514 | ORF; ORF | CAGCGUGCUGCGCGCCAACGACGUGCUGUGG |
| 16 | 2 | 440, 500 | ORF; ORF | GGCCCCGGCCCCGGCA |
| 13 | 2 | 316, 358 | ORF; ORF | CCUGGCCGCCUAC |
| 12 | 2 | 13, 38 | 5′ region; 5′ region | AAUCAAUCAUCA |
| 12 | 2 | 548, 764 | ORF; ORF | AGCCUGACCGCC |
